# The Dilute domain in Canoe is not essential for linking cell junctions to the cytoskeleton but supports morphogenesis robustness

**DOI:** 10.1242/jcs.261734

**Published:** 2024-03-21

**Authors:** Emily D. McParland, T. Amber Butcher, Noah J. Gurley, Ruth I. Johnson, Kevin C. Slep, Mark Peifer

**Affiliations:** ^1^ Department of Biology, University of North Carolina at Chapel Hill, CB#3280, Chapel Hill, NC 27599-3280, USA; ^2^ Biology Department, Wesleyan University, Middletown, CT 06459, USA; ^3^ Lineberger Comprehensive Cancer Center, University of North Carolina at Chapel Hill, Chapel Hill, NC 27599, USA

**Keywords:** Afadin, Canoe, *Drosophila*, Adherens junction, Cadherin, Morphogenesis

## Abstract

Robust linkage between adherens junctions and the actomyosin cytoskeleton allows cells to change shape and move during morphogenesis without tearing tissues apart. The *Drosophila* multidomain protein Canoe and its mammalian homolog afadin are crucial for this, as in their absence many events of morphogenesis fail. To define the mechanism of action for Canoe, we are taking it apart. Canoe has five folded protein domains and a long intrinsically disordered region. The largest is the Dilute domain, which is shared by Canoe and myosin V. To define the roles of this domain in Canoe, we combined biochemical, genetic and cell biological assays. AlphaFold was used to predict its structure, providing similarities and contrasts with Myosin V. Biochemical data suggested one potential shared function – the ability to dimerize. We generated Canoe mutants with the Dilute domain deleted (CnoΔDIL). Surprisingly, they were viable and fertile. CnoΔDIL localized to adherens junctions and was enriched at junctions under tension. However, when its dose was reduced, CnoΔDIL did not provide fully wild-type function. Furthermore, *canoe*Δ*DIL* mutants had defects in the orchestrated cell rearrangements of eye development. This reveals the robustness of junction–cytoskeletal connections during morphogenesis and highlights the power of natural selection to maintain protein structure.

## Teaser

**Highlighted Article:** Cell junctions link cells and anchor the cytoskeleton. Here, we define the role in this process of the conserved Dilute domain in the complex multidomain Canoe protein (a homolog of afadin).

## INTRODUCTION

Building the architecture of tissues and organs requires individual cells to work together, changing shape and moving in coordinated ways. Cell shape change requires force to be exerted on the plasma membrane. This occurs at cell–cell adherens junctions (AJs) and cell–extracellular matrix junctions. At these junctions transmembrane cadherins or integrins connect cells to one another or to the extracellular matrix, respectively. Their cytoplasmic domains then organize linker proteins that connect to the actin cytoskeleton on which myosin motor proteins walk, generating the force required to drive shape change.

Scientists studying integrin-based adhesions have long appreciated the complexity of the mechanosensitive protein network linking integrin cytoplasmic tails to the actomyosin cytoskeleton, with dozens of components arranged in distinct layers (Case and Waterman, 2015). A cell biologist's view of the linkage of cadherins to the cytoskeleton started much more simply, with a picture of a linear and direct linkage. In this picture, β-catenin bound both the cadherin tail and α-catenin, while α-catenin bound F-actin. Subsequent work has revealed that this picture is significantly over-simplified (reviewed in Perez-Vale and Peifer, 2020; Yap et al., 2018). We now recognize that a much larger network of proteins mediates this linkage, including *Drosophila* Canoe (Cno; the homolog of mammalian homolog afadin; hereafter Cno/afadin when referred to collectively), Polychaetoid (the homolog of mammalian ZO-1, also known as TJP1), Ajuba, Vinculin, Sidekick and likely others. This network provides redundancy and thus robustness, with some protein interactions and even some individual proteins dispensable for baseline function. The protein network is also mechanically sensitive, with the linkage strengthened by pulling force on AJs.

**Fig. 1.**
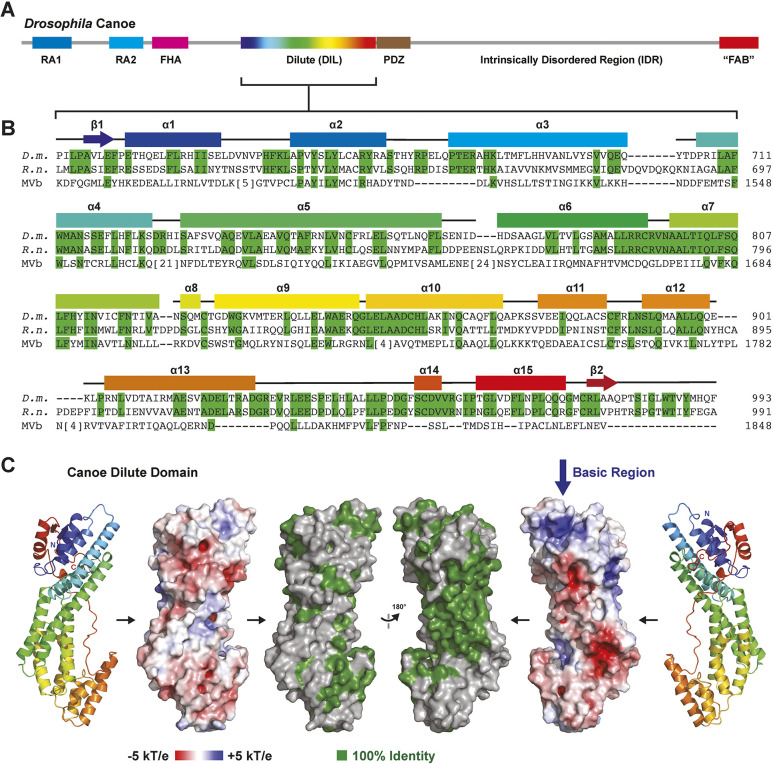
**The Cno/afadin family DIL domain has a conserved central groove.** (A) Domain architecture of *Drosophila* Cno. The DIL domain is colored in a blue-to-red spectrum, representative of the secondary structure color codes in B and C. (B) Sequence alignment of *Drosophila melanogaster* (D.m.) Cno, *Rattus norvegicus* (R.n.) afadin, and the sequence of the human MyoVb (MVb) DIL domain. Residues identical between Cno and afadin are highlighted in green, as are the corresponding residues in MyoVb to which the identity extends. Predicted secondary structure elements of the Cno AlphaFold prediction (Jumper et al., 2021; Varadi et al., 2022) are shown above the alignment. (C) AlphaFold structure prediction of the Cno DIL domain shown (from left to right) in cartoon format, space-filling format showing electrostatics, and conservation (residues identical between rat afadin and fly Cno as highlighted in B), followed by views of each after a 180° rotation about the *y*-axis.

Part of the robustness of the network is conferred by the fact that many proteins in it are large multidomain proteins that can bind many partners. We use Cno and its mammalian homolog Afadin as a model for exploring how this complex protein structure confers function. Cno and Afadin share five predicted folded protein domains, followed by a long intrinsically disordered region (IDR) (Fig. 1A; Gurley et al., 2023). At their N-terminus are two Ras-association (RA) domains, which bind the small GTPase Rap1 (Boettner et al., 2000). Rap1 ‘activates’ Cno by mechanisms we are only starting to understand (Boettner et al., 2003; Bonello et al., 2018; Perez-Vale et al., 2023). The central PDZ domain binds several partners, including the transmembrane junctional proteins E-cadherin (Ecad; Sawyer et al., 2009) and Echinoid (nectins in mammals) (Takahashi et al., 1999; Wei et al., 2005). Between the RA and PDZ domains are two other domains – the Forkhead-associated (FHA) and Dilute (DIL) domains – which can be recognized by sequence, but the biochemical function of which remain unclear. The C-terminal IDR carries one or more F-actin-binding sites, the most C-terminal of which is referred to as the F-actin-binding (FAB) region (Mandai et al., 1997; Sakakibara et al., 2018).

We are using the powerful genetic tools available in *Drosophila* to take apart this complex multidomain machine and analyze the function of different domains. Cno is important for almost every cell shape change or morphogenetic movement in the embryo, ranging from initial positioning of AJs (Choi et al., 2013) to apical constriction of mesodermal cells (Sawyer et al., 2009), convergent elongation of the body axis (Sawyer et al., 2011; Yu and Zallen, 2020), and the collective cell migrations of dorsal closure and head involution (Boettner et al., 2003; Choi et al., 2011). Cno is required to strengthen junction–cytoskeletal connections at AJs under elevated force, and in its absence cytoskeletal–AJ connections are disrupted. We previously generated a CRISPR-based tool that allows us to replace the endogenous *cno* coding sequence with GFP-tagged mutant proteins (Perez-Vale et al., 2021). Deleting both N-terminal RA-domains nearly eliminated Cno function (Perez-Vale et al., 2021). CnoΔRA localization to forming AJs was altered, although this was restored in later stages. However, the tension-sensitive recruitment of Cno to AJs under tension requires the RA domains. We also explored the roles of the PDZ domain and FAB region (Perez-Vale et al., 2021), hypothesizing they would be essential for function by linking Ecad to actin. However, to our surprise, both were dispensable for viability. Sensitized assays revealed that these domains reinforce AJs under tension. Together, these data and those from others support the idea that a robust AJ–cytoskeletal linkage is conferred by multivalent interactions.

Here, we focus on the DIL domain of Cno, asking what role(s) it plays in Cno function. The DIL domain was first identified in the unconventional myosin, myosin V (MyoV), which transports vesicles (reviewed in Wong and Weisman, 2021). The DIL domain of MyoV (also known as the cargo-binding domain or globular tail domain) provides the cargo-binding site. It has an elongated structure in which 15 amphipathic α-helices are connected by short and long loops (Fig. 1B), and binds many partners and has multiple functions. It binds the MyoV motor domain to act as an auto-inhibitor of motor activity (Niu et al., 2022). It is also the docking site for multiple cargo-specific adapter proteins, including melanophilin, Spire2, molecules interacting with CasL (MICAL1) and Rab-interacting lysosomal protein-like 2 (RILPL2). These proteins dock on three spatially distinct regions on the DIL domain (e.g. Wei et al., 2013). Small Rab family GTPases also dock on the DIL domain (Pylypenko et al., 2013). Structural studies suggest that some adapters can indirectly lead to dimerization of MyoV via their DIL domain interaction (Wei et al., 2013). At least a subset of MyoV DIL domains can also dimerize directly, with two different potential modes of interaction identified (e.g. Nascimento et al., 2013; Zhang et al., 2016). Thus, in the protein family where it has been most closely examined, the DIL domain mediates many functions, serving as a hub for both intra- and inter-molecular interactions.

The only other proteins known to have DIL domains are the Cno/afadin family, found in all animals, and their distantly related and less studied vertebrate paralogs RADIL and Rasip. The DIL domains of human afadin and *Drosophila* Cno share 48% amino acid sequence identity (Gurley et al., 2023). Conservation in the vertebrate lineage is even stronger, with the DIL domains of human and zebrafish afadin sharing 89% identity. Even the distant animal relative Trichoplax contains a Cno/afadin relative, with a DIL domain 38% identical to that in *Drosophila*. Despite this strong conservation, little is known about the molecular or biological functions of the DIL domain in either mammals or *Drosophila*. Yeast two-hybrid protein interaction screens have identified the coiled-coil protein afadin DIL domain-interacting protein (ADIP; also known as SSX2IP) as a binding partner of afadin that can interact with its DIL domain (Asada et al., 2003). ADIP can regulate migration of cultured cells, by regulating the small GTPase Rac (Fukumoto et al., 2011). Only two tests of afadin DIL domain function have been reported, both in cell culture. In migrating mammalian cells, an afadin mutant lacking the DIL domain did not fully support cell migration in a cultured cell wounding assay, matching the effect of ADIP knockdown (Fukumoto et al., 2011). In cultured MDCK cells manipulated to elevate junctional tension, an afadin mutant lacking the DIL domain provided strong rescue of the gaps in cell junctions under tension caused by afadin knockdown but did not fully rescue cell shape (Choi et al., 2016). However, this latter cell culture assay used afadin knockdown, not knockout, and thus in this system even an afadin mutant lacking the Rap1-binding RA domains, which provides only minimal function in *Drosophila*, provided significant rescue (Choi et al., 2016). Here, to fully explore the biochemical and biological function of the DIL domain in the diverse roles of Cno, we combined biochemical, genetic and cell biological assays.

## RESULTS

### The DIL domains of Cno/afadin are predicted to have a unique, conserved central groove

The DIL domains of Cno and afadin are well conserved in sequence and length, with 47% of the 374 fly amino acids identical to the corresponding rat 393 amino acids (Gurley et al., 2023; Fig. 1B). The structure of the DIL domain has been solved from multiple MyoV family members, both alone and in complex with binding partners, but the structure of Cno/afadin DIL domains has not been experimentally determined. New tools made available by the development of AlphaFold have allowed us to examine the predicted structures of the DIL domain in Cno and afadin (Fig. 1B,C; Fig. S1A–C; Jumper et al., 2021; Varadi et al., 2022). Overall, the AlphaFold Cno and afadin DIL domain structural models have high confidence. The predicted local distance difference test (pLDDT) is a per-residue estimate of its confidence on a scale from 0–100. The Cno DIL pLDDT range is 99–62, with Cα average of 93. The afadin DIL pLDDT range is 97–49, with Cα average of 90. Low confidence regions map to loops, which in the MyoVb DIL crystal structure (PDB: 4J5M) correspond to disordered segments or loops with relatively high B-factors (Fig. S1A–C; Nascimento et al., 2013). The DIL domain of Cno is predicted to contain 15 α-helices that, as in the MyoV DIL domain, form an elongated domain. A C-terminal tail is predicted to fold back along the domain, positioning the C-terminal β2 strand anti-parallel to the N-terminal β1 strand. The region containing the N- and C-termini, which are predicted to be proximal to one another, is predominantly basic, as compared to the rest of the domain, which is primarily acidic (Fig. 1C). The PDZ domain, which is just C-terminal to the DIL domain, binds membrane-associated proteins. Thus, the basic nature of the DIL domain proximal to the PDZ domain might serve to complement the negatively charged plasma membrane.

Aligning the predicted structures of Cno and afadin with the solved structure of MyoVb (Nascimento et al., 2013) highlights unique features of the Cno/afadin family as well as regions where either Cno or afadin is more like MyoVb whereas the other deviates (Fig. S1D–G). One area involves the terminal regions of the domain (Fig. S1D). Although Cno and afadin are predicted to have similar structure over this region (Fig. S1D, left panel), MyoVb lacks the α15 helix (Fig. S1D, left panel, red arrow) and has a larger insertion between α5 and α6 that extends away from the helices and occupies the site corresponding to the Cno/afadin C-terminal tail (Fig. S1D, right panel, lower red arrow). In Cno/afadin, the corresponding loop occurs between the predicted helices α4 and α5 and is minimal in length. The structural alignment also highlights predicted loops of different lengths and positioning, including (i) the Cno α2–α3 loop, which is similar to afadin (Fig. S1D, left panel), but extended relative to MyoVb (Fig. S1D, right panel, upper red arrow), (ii) the Cno α3–α4 loop (Fig. S1E, red arrow), which is predicted to be similar to MyoVb but distinct from that of afadin, (iii) the Cno α5–α6 loop, which is predicted to be extended in afadin (Fig. S1F, red arrow) and disordered in MyoVb (Fig. S1F, the MyoV phospho loop, red circles and red dotted line), suggesting high structural plasticity, and (iv) the Cno α12–α13 loop, which is similar between the afadin model and the MyoVb structure but is shorter and distinctive in the Cno model (Fig. S1G, lower red arrow). The predicted α13 helices of Cno and afadin are similar, but the corresponding MyoVb helix is truncated from the C-terminal region (Fig. S1G, upper red arrow). Collectively, the structural comparisons highlight a common core domain with structural variations primarily focused on the length and positioning of select loops. Many of these loops have relatively lower prediction confidence scores in the AlphaFold models (Fig. S1A,B) or higher B-factors (or could not be modeled) in the MyoVb crystal structure (Fig. S1C; Nascimento et al., 2013).

We also examined which regions of Cno and afadin are most highly conserved. Strong sequence identity between Cno and afadin maps to a predicted central groove on the domain, largely consisting of residues from α6, α7, α9 and α10 that are distinct from those in MyoV (Fig. 1B,C). This suggests a potential conserved site specific to the Cno/afadin family for molecular interactions.

**Fig. 2.**
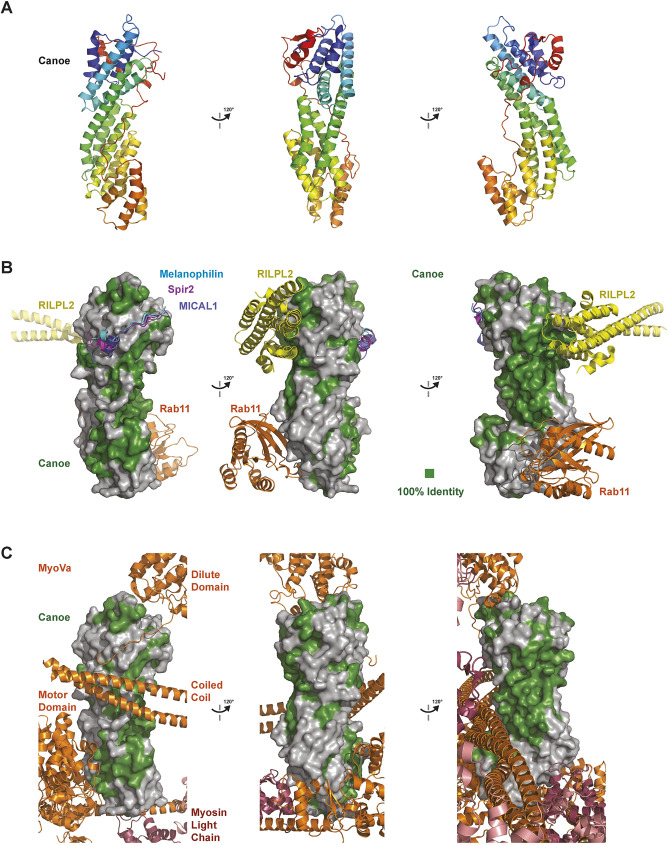
**The predicted Cno/afadin DIL domains share a conserved central groove that is not engaged by MyoV-binding factors in MyoV complex structures determined to date.** (A) The AlphaFold Cno DIL domain predicted structure, shown in cartoon format in consecutive 120° rotations about the *y*-axis, colored as in Fig. 1C. (B) Comparative analysis of where MyoV-binding proteins bind the MyoV DIL domain relative to conserved residues of the Cno/afadin family DIL domain. MyoV-binding proteins shown do not fully engage the central groove of the DIL domain that is highly conserved in Cno/afadin. Cno DIL domain oriented as in A, shown in surface representation, with residues identical between *Drosophila* (D.m.) Cno and *R. norvegicus* (R.n.) afadin colored green. MyoV-binding proteins: RILPL2 (yellow, PDB 4KP3; citations for structures are in the Materials and Methods), Rab11 (orange, PDB 4LX0), Melanophilin (cyan, PDB 4KP3), Spire2 (Spir2; purple, PDB 5JCY) and MICAL1 (dark blue, PDB 6KU0) are shown after structurally aligning the respective MyoV DIL domain from the complex with the Cno DIL domain. The MyoV DIL domain from each complex structure is not shown. (C) Comparative analysis of how domains of the full-length homodimeric MyoVa protein (the motor domain, coiled coil and DIL domain) and myosin light chains engage the DIL domain of the motor relative to conserved residues of the Cno/afadin family DIL domain. Cno DIL domain oriented as in A, shown in surface representation, with residues identical between D.m. Cno and R.n. afadin colored green. MyoVa domains (orange) and myosin light chains (pink and red; from PDB 7YV9), are shown after structurally aligning one of the MyoVa DIL domains and the Cno DIL domain. The aligned MyoVa DIL domain is not shown, but the non-aligned MyoVa DIL domain of the homodimer is shown. MyoVa interdomain interactions that involve the DIL domain do not fully engage the central groove of the DIL domain that is highly conserved in Cno/afadin.

Although the conserved central groove of the Cno/afadin DIL domain is distinct from MyoV, we inquired whether any MyoV-binding partners for which structures have been determined, occupied binding sites that overlapped the central groove that is conserved between Cno and afadin (Fig. 2). We structurally aligned MyoV DIL domains in complex with a variety of binding partners (melanophilin, Spire2, MICAL1, RILPL2 and Rab11; Niu et al., 2020; Pylypenko et al., 2013; 2016; Wei et al., 2013) with the predicted Cno structure (Jumper et al., 2021; Varadi et al., 2022). Modeling the sites occupied by these MyoV-binding partners on the predicted Cno DIL domain structure and comparing these to regions of Cno/afadin sequence conservation revealed that none of these MyoV-binding factors fully engage the predicted conserved central groove of Cno (Fig. 2B). A region of the RILPL2-binding site partially overlaps with the conserved central groove but leaves most of the groove open (Fig. 2B, right panel). Rab11 and the peptide-binding modes of melanophilin, Spire2, and MICAL1 engage the MyoV DIL domain in regions that are not conserved between Cno and afadin. The MyoV DIL domain is part of a large myosin motor complex, in which intramolecular interactions maintain the motor in an off state. We next examined how Cno/afadin conservation compares to regions involved in the interdomain interactions involving the DIL domain of MyoV (Fig. 2C). In the full-length MyoVa homodimer structure, the MyoVa DIL domain engages the motor domain, the coiled coil, and the sequence N-terminal to the DIL domain of the homodimeric mate. The latter interaction occurs in a mode akin to the melanophilin, Spire2 and MICAL1 peptides (Niu et al., 2022). None of these interdomain interactions engage the central groove except for the coiled coil, which has a partial binding site overlap, but leaves most of the groove accessible (Fig. 2C).

### The DIL domain of Cno can dimerize *in vitro*

**Fig. 3.**
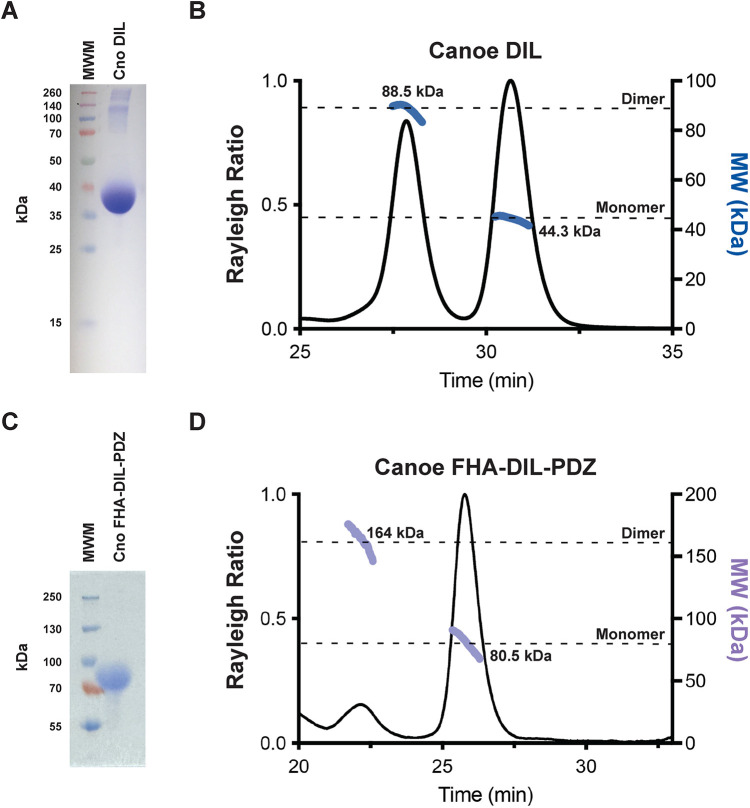
**The Cno DIL domain can homodimerize *in vitro*.** (A) SDS-PAGE gel analysis of the purified Cno DIL domain (aa 613–1006), with molecular mass 44.5 kDa, which runs anomalously at ∼37 kDa. A small amount of contaminants, greater than 120 kDa in size, are present. (B) SEC-MALS analysis of the Cno DIL domain (aa 613–1006) shows both a monomeric and a dimeric population. Rayleigh ratio (left *y*-axis, solid black line) and experimentally determined molecular mass (right y-axis, blue lines) are indicated relative to elution time (*x*-axis). The formula mass of a monomer and a homodimer are indicated with dashed black lines. 27% of the injected DIL domain eluted as a homodimer (left peak), whereas 73% eluted as a monomer (right peak). (C) SDS-PAGE gel analysis of purified Cno FHA-DIL-PDZ protein (aa 372–1110), with molecular mass 81.5 kDa. (D) SEC-MALS analysis of the Cno FHA-DIL-PDZ protein (aa 372-1110) shows both a monomeric and a dimeric population. Rayleigh ratio (left *y*-axis, solid black line) and experimentally determined molecular mass (right *y*-axis, purple lines) are indicated relative to elution time (*x*-axis). The formula mass of a monomer and a homodimer are indicated with dashed black lines. 7% of the injected FHA-DIL-PDZ protein eluted as a homodimer (left peak), whereas 93% eluted as a monomer (right peak). Images in this figure are representative of biological duplicates.

Data from both mammalian afadin and *Drosophila* Cno suggest that these proteins can dimerize or oligomerize (Bonello et al., 2018; Mandai et al., 1997). The structural similarities with the DIL domain of MyoV, which can dimerize, encouraged us to explore whether the DIL domain of Cno shared the ability to dimerize. To test this, we produced the DIL domain of Cno in *Escherichia coli* and purified it (Fig. 3A). We then used size exclusion chromatography and multi-angle light scattering (SEC-MALS) to determine the molecular mass of the Cno DIL domain. Strikingly, the Cno DIL domain migrated as both a monomer and a dimer (Fig. 3B). This suggests that one function of the DIL domain might be to mediate Cno dimerization. To determine whether dimerization was retained in proteins carrying additional folded domains, we cloned a construct extending from the beginning of the Cno FHA domain to the end of the PDZ domain (FHA-DIL-PDZ), expressed this in *E. coli* and purified it (Fig. 3C). This construct also migrated as both a monomer and a dimer (Fig. 3D), although the amount in the dimer fraction was lower. It will be important in the future to explore potential roles for the DIL domain in the oligomerization we and others have observed *in vivo*.

### Creating a mutant to test the function of DIL domain of Cno in *Drosophila*

**Fig. 4.**
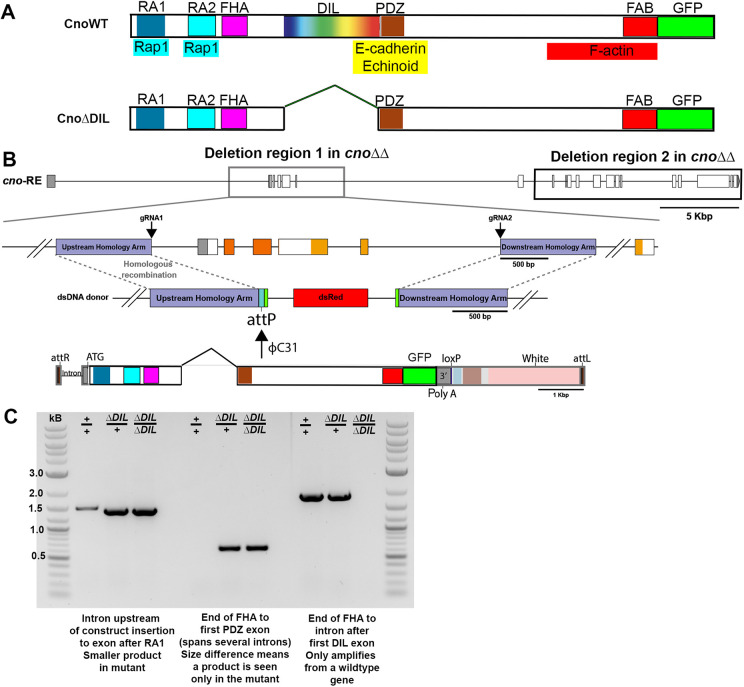
**Generating a mutant to assess the function of the Cno DIL domain.** (A) Diagram of the Cno protein and the CnoΔDIL mutant. (B) Strategy for generating *cno*Δ*DIL.* We started with the *cno*ΔΔ chromosome, which has most of the *cno* coding sequence deleted and has an attP site near the 3′ end of the first intron (Perez-Vale et al., 2021). The modified *cno* coding sequence with the DIL domain deleted and a C-terminal GFP-tag added was inserted in place of the second-fifth exons of *cno* using site-specific recombination via phiC31 integrase. (C) PCR reactions confirming the correct mutation; see Materials and Methods for details (image representative of at least two repeats).

We next turned to testing the DIL domain function *in vivo* by precisely deleting it from Cno protein. We designed our mutant guided by the AlphaFold predictions of domain structure (Fig. 1C), deleting amino acids 613–993 of *Drosophila* Cno. Our deletion starts in a poorly conserved region, predicted to be disordered, 11 amino acids N-terminal to the sequence we defined as the DIL domain and ended five amino acids before the C-terminal end of the DIL domain to avoid inadvertent effects on the adjacent PDZ domain (Fig. 4A). We then used a system we established in *Drosophila* that allows us to place GFP-tagged versions of the *cno* gene, wild-type or with site-directed mutants, into a modified version of the *cno* locus, in which most of the *cno* protein coding sequence is deleted (the ΔΔ background; Fig. 4B; Perez-Vale et al., 2021). We inserted the modified coding sequence with the DIL domain deleted in place of the second exon of *cno*, using site-specific recombination via phiC31 integrase (Fig. 4B; Bischof et al., 2007) – the inserted sequence also carries the *white* (*w*) gene as a selectable marker. Thus, inserted *cno* transgenes are expressed under endogenous gene control at the right times and places. Insertion of a GFP-tagged wild-type Cno fully rescued viability and fertility (Perez-Vale et al., 2021). We verified the accuracy of our genetic modifications with PCR amplification of the *cno*Δ*DIL* genomic locus from transgenic *Drosophila*, using primer pairs that distinguished the wild-type and altered gene (Fig. 4C), and sequenced the amplified product across the span of the deletion. We refer to this mutant as *cno*Δ*DIL*.

### CnoΔDIL protein accumulates at levels that are similar to wild-type Cno

**Fig. 5.**
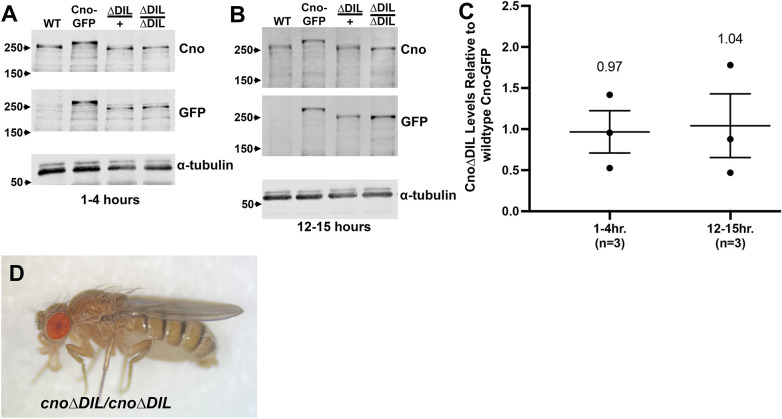
**CnoΔDIL protein accumulates at normal levels and *cno*Δ*DIL* mutants are viable.** (A,B) Embryonic protein extracts of the indicated ages, immunoblotted with antibodies to Cno, GFP or α-tubulin as a loading control. *cnoWT-GFP* embryos were a positive control for GFP antibody. Because of the deletion, GFP-tagged CnoΔDIL protein runs at a similar apparent molecular mass to wild-type Cno. (C) Calculated levels of CnoΔDIL relative to wild-type Cno. Three replicates (dots) are shown, with the broad line illustrating the mean value and the narrower bands illustrating the s.e.m. (D) Homozygous *cno*Δ*DIL* mutant (adults are ∼3 mm long).

Our previous site-directed Cno mutants accumulated at levels similar to those of wild-type Cno (Perez-Vale et al., 2021). CnoΔDIL is recognized by both our standard anti-Cno antibody, which recognizes a region in the IDR (Sawyer et al., 2009), and with antibodies to the C-terminal GFP tag. We generated embryonic protein extracts from both early (1–4 h) or mid-stage (12–15 h) embryos and examined them by immunoblotting with anti-Cno and anti-GFP antibodies. We used antibodies to alpha-tubulin as a loading control. We examined stocks either heterozygous or homozygous for *cno*Δ*DIL*, with wild-type endogenous Cno and GFP-tagged wild-type Cno, which accumulates at wild-type levels (Perez-Vale et al., 2021), as standards. CnoΔDIL accumulated at levels similar to wild-type endogenous Cno and to GFP-tagged wild-type Cno in both early and mid-stage embryos (Fig. 5A,B). Quantifying protein levels relative to GFP-tagged wild-type Cno using multiple samples verified that CnoΔDIL accumulated at essentially wild-type levels (Fig. 5C), validating its use to examine Cno DIL domain function.

### Deleting the Cno DIL domain does not compromise viability or fertility

*cno*-null mutants are zygotically embryonic lethal (Jürgens et al., 1984; Sawyer et al., 2009). The Rap1-binding RA domains of Cno are essential for viability, whereas the PDZ domain and the C-terminal FAB region are dispensable for viability and largely or completely dispensable for fertility (Perez-Vale et al., 2021). We thus assessed whether the conserved DIL domain is essential for viability. After obtaining *cno*Δ*DIL* flies, we outcrossed them to a wild-type stock (carrying the *y* and *w* mutations), selecting for the *w^+^* gene introduced into the *cno* locus*.* After multiple generations of outcrossing, we created a balanced stock using a third chromosome balancer chromosome and examined whether homozygous *cno*Δ*DIL* adults could be obtained. We obtained homozygous adults (Fig. 5D) and created a homozygous mutant stock, which we once again verified by PCR (Fig. 4C) and by western blotting (Fig. 5A,B). We saw no significant embryonic lethality of zygotic mutant embryos (2% lethality; *n*=497 embryos). In our earlier analyses, we found that although *cno*Δ*FAB* flies are viable and fertile, embryonic viability of maternal and zygotic mutants is reduced (Perez-Vale et al., 2021). We thus also assessed whether this was true for *cno*Δ*DIL.* We also saw no significant embryonic lethality of maternal and zygotic mutants (6%; *n*=199; this is within the range of lethality of wild-type flies). Thus, deleting the DIL domain of Cno does not compromise viability or fertility.

### CnoΔDIL protein localizes to and supports initial assembly of AJs

We next assessed the role of the DIL domain in Cno protein localization and looked closely at developing embryos to determine whether there were defects in AJ assembly and maintenance or cell shape changes subtle enough to be compatible with viability. Cno localizes to embryonic AJs from their initial assembly (Bonello et al., 2018; Sawyer et al., 2009). We visualized CnoΔDIL using either the GFP tag or, in homozygous or hemizygous mutants, antibodies to Cno (images shown include embryos maternally and zygotically mutant for *cno*Δ*DIL* or embryos transheterozygous for *cno*Δ*DIL* and our protein-null allele *cno^R2^*).

**Fig. 6.**
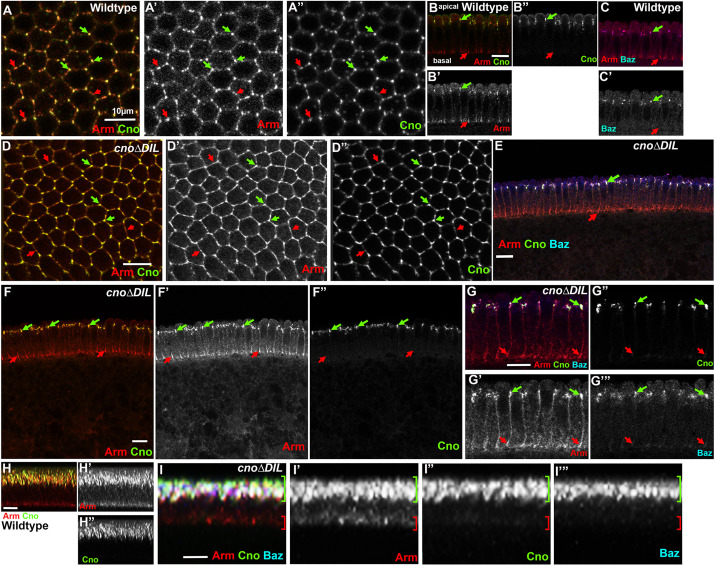
**CnoΔDIL localizes and functions correctly as AJs assemble during cellularization.** Late cellularization *Drosophila* embryos, antigens and genotypes indicated. (A,D) Apical junction views of wild-type (A) or maternal and zygotic *cno*Δ*DIL* mutant embryos (D). In wild type, Arm localizes to all spot AJs (A, red arrows), with some enrichment at TCJs (A, green arrows), whereas Cno is substantially enriched at TCJs. CnoΔDIL protein localizes normally, with strong enrichment at TCJs (D, green versus red arrows). (B,C,E–G) Cross sections, apical up. (B,C) In wild type, Arm localizes to both nascent apical junctions (green arrows) and basal junctions (red arrows), whereas Cno and Baz are restricted to apical junctions. (E–G) In maternal and zygotic *cno*Δ*DIL* mutant embryos Arm and Baz localize normally, and CnoΔDIL is properly localized to apical junctions (green arrows versus red arrows). (H,I) MIPs of of multiple cross sections. This imaging method emphasizes the enrichment of Arm, Cno, and Baz to apical junctions in wild-type (H) and confirms that CnoΔDIL retains normal localization and function during cellularization (I) (green and red brackets). Scale bars: 10 µm. Images in this figure representative of six embryos examined at this stage.

We first examined Cno localization and function as AJs assemble during cellularization. The core AJ complex, including Armadillo (Arm; the fly β-catenin), localized to spot AJs that assemble near the apical end of the invaginating membrane and are positioned around the apical perimeter of forming cells (Harris and Peifer, 2004); Fig. 6A′,B′, green arrows), with some modest enrichment in tricellular junctions (TCJs; Fig. 6A,A′, green arrows). The cadherin–catenin complex also localized along the lateral cell membrane and was enriched in basal junctions at the tip of the invaginating membrane (Fig. 6B′ red arrow). Both wild-type Cno and Bazooka (Baz; the fly Par3) localized with Arm in forming apical junctions (Fig. 6A″,B″ green arrows; Fig. 6C,C′ green arrows), but were absent from basal junctions (Fig. 6A″,B″ red arrows; Fig. 6C,C′ red arrows). At this stage, Cno was significantly enriched in TCJs relative to the bicellular spot AJs (Fig. 6A,A″, green versus red arrows; Bonello et al., 2018). During cellularization Cno is required for correct initial positioning of AJs – in its absence both Arm and Baz are no longer enriched in nascent junctions and instead localize all along the lateral membrane (Choi et al., 2013).

We thus examined CnoΔDIL localization and function during cellularization. Like wild-type Cno, CnoΔDIL localized to spot AJs but was more substantially enriched at TCJs relative to Arm (Fig. 6D,D″, red versus green arrows). Along the *Z*-axis, CnoΔDIL remained localized along with Arm to nascent apical junctions (Fig. 6E,F,G green arrows) as membranes invaginated, while Arm also localized to the basal junctions at the leading edge of the invaginating membrane (Fig. 6E,F,G, red arrows). Baz also remained enriched in nascent apical junctions in *cno*Δ*DIL* mutants (Fig. 6G‴). Correct apical enrichment of all three proteins in the wild type is emphasized in maximum X/Z intensity projections of multiple cells (Fig. 6H), and similar projections revealed parallel apical enrichment in *cno*Δ*DIL* mutants (Fig. 6E). Thus, CnoΔDIL retains normal localization and function during cellularization – in these properties it resembled CnoΔFAB and CnoΔPDZ but differed from CnoΔRA (Perez-Vale et al., 2021).

### CnoΔDIL is correctly enriched at AJs under elevated tension and supports morphogenesis

**Fig. 7.**
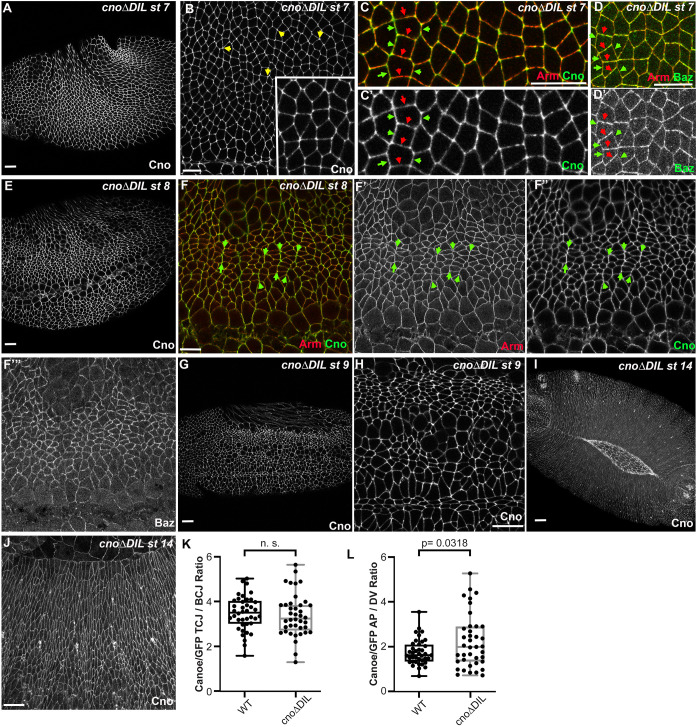
**CnoΔDIL localizes and functions correctly during embryonic morphogenesis.** Maternal and zygotic *cno*Δ*DIL* mutant embryos, anterior left, dorsal up, stages, genotypes and antigens indicated. (A–D) Stage 7 embryos. (A,B) CnoΔDIL localizes to AJs (A) and is enriched at TCJs (B, arrows and inset). (C) CnoΔDIL is slightly enriched at aligned AP borders (green arrows) versus DV borders (red arrows). AJ defects are not observed. (D) Baz remains localized all around the cells with some polarization to DV borders, rather than exhibiting the accentuated planar polarity seen in *cno*-null mutants. (E,F) Stage 8. CnoΔDIL continues to localize to AJs (E), with some enrichment at aligned AP borders (F″, arrows). AJ defects are not observed (F′) and Baz continues to localize all around the cell (F‴). (G–J) CnoΔDIL continued to localize to AJs at stage 9 (G,H) and during dorsal closure (I,J) and no defects in morphogenesis were observed. (K) Quantification of enrichment of wild-type Cno or CnoΔDIL at TCJs. Statistical significance was calculated by Welch's unpaired two-tailed *t*-test or Brown Forsythe and Welch ANOVA test. (L) Quantification of enrichment of wild-type Cno or CnoΔDIL at aligned AP versus DV cell borders. For both box-and-whisker graphs, box shows the 25th–75th percentile, the whiskers show 5th–95th percentiles, the horizontal line shows the median and the plus sign (+) is the mean. Statistical significance was calculated using a one-way ANOVA. Scale bars: 10 µm.

As gastrulation begins, the germband elongates along the anterior–posterior (AP) axis and narrows in the dorsal–ventral (DV) axis. Consistent with the viability of *cno*Δ*DIL* mutants, germband extension proceeded without apparent defects, and like endogenous Cno, CnoΔDIL remained localized to the AJ as they matured (Fig. 7A,E). During this process, AJ and cytoskeletal proteins become planar polarized across the epithelium. Myosin and F-actin become enriched at AP cell borders, forming contractile cables that constrict those boundaries and rearrange cells (reviewed in Perez-Vale and Peifer, 2020). Meanwhile, Baz, Ecad, Arm and Polychaetoid (Pyd; fly ZO-1) become enriched at DV cell borders, opposite to myosin. At stage 7 wild-type Cno is slightly enriched at AP borders relative to DV borders (Sawyer et al., 2011), similar to myosin, and this enrichment is also apparent along aligned AP borders at stage 8, as more dorsal cells round up to divide. Cno also remains enriched at TCJs during germband extension (Perez-Vale et al., 2021). This Cno enrichment at TCJs is a response to elevated tension (Yu and Zallen, 2020). We thus looked at CnoΔDIL localization, examining enrichment at TCJs and AP borders. CnoΔDIL remained clearly enriched at TCJs (Fig. 7B, arrows); quantification revealed its enrichment there was similar to that of wild-type Cno (Fig. 7K). Similarly, CnoΔDIL retained a slight enrichment at AP borders at stage 7 (Fig. 7C, green versus red arrows) and along aligned AP borders at stage 8 (Fig. 7F, green arrows). Quantification confirmed this subtle enrichment at aligned AP borders (Fig. 7L). Once again, these properties of CnoΔDIL were similar to those of CnoΔFAB and CnoΔPDZ but different from those of CnoΔRA, which lost enrichment at TCJs and reversed enrichment to become elevated at DV rather than AP borders (Perez-Vale et al., 2021). Cno function is also crucial to restrain Baz planar polarity. In its absence Baz is virtually lost at AP borders and thus becomes highly enriched at DV borders, and often is restricted to the central region of those borders (Sawyer et al., 2011). In contrast, in *cno*Δ*DIL* mutants Baz localized all around cells at stages 7 and 8, with moderate enrichment on DV borders (Fig. 7D, red versus green arrows; Fig. 7F‴).

We also looked at CnoΔDIL localization after the completion of germband extension, during stage 9 (Fig. 7G,H), and during dorsal closure (stage 14; Fig. 7I,J) and saw no differences from the localization of wild-type Cno and no deviations from normal progression of development. Taken together, these data reveal that *cno*Δ*DIL* mutants retain full function in positioning nascent AJs during cellularization, in supporting cell shape changes and protein planar polarity during germband extension, and in completing dorsal closure, all events known to require Cno function. Furthermore, CnoΔDIL protein localizes to AJs and is correctly enriched at cell junctions under elevated tension.

### Deleting the DIL domain reduces Cno function when Cno protein levels are reduced

Given the strong conservation of the DIL domain in Cno/afadin relatives in distantly related animals, we were surprised at its apparent dispensability. In our earlier analysis of *cno*Δ*PDZ* and *cno*Δ*FAB* mutants, we tested protein function in a sensitized situation in which we reduced levels of the mutant proteins. To do so, we made each mutant heterozygous with our canonical protein-null *cno* allele, *cno^R2^* (Sawyer et al., 2009). *cno^R2^* is zygotically embryonic lethal, so in a cross of *+/cno^R2^* parents, the 25% of embryos who are *cno^R2^/cno^R2^* die as embryos, whereas the *+/cno^R2^* and *+/+* progeny from this cross are fully viable. However, due to the strong maternal contribution of wild-type Cno, although *cno* maternal and zygotic mutants have defects in most morphogenetic movements, most *cno^R2^* homozygous null zygotic mutants only have mild defects in head involution but few defects in other morphogenetic events like dorsal closure (Gurley et al., 2023; Sawyer et al., 2009). This creates a sensitized situation where small reductions in maternal Cno function can enhance these defects. Replacing wild-type Cno with either *cno*Δ*PDZ* or *cno*Δ*FAB* substantially enhances the zygotic cuticle phenotype of *cno^R2^* homozygous zygotic mutants and leads to reduced viability of *cno*Δ*PDZ/cno^R2^* or *cno*Δ*PDZ/cno^R2^* progeny (Perez-Vale et al., 2021), revealing that CnoΔFAB and CnoΔPDZ do not provide fully wild-type function. We thus implemented this sensitized genetic test to examine *cno*Δ*DIL.*

**Fig. 8.**
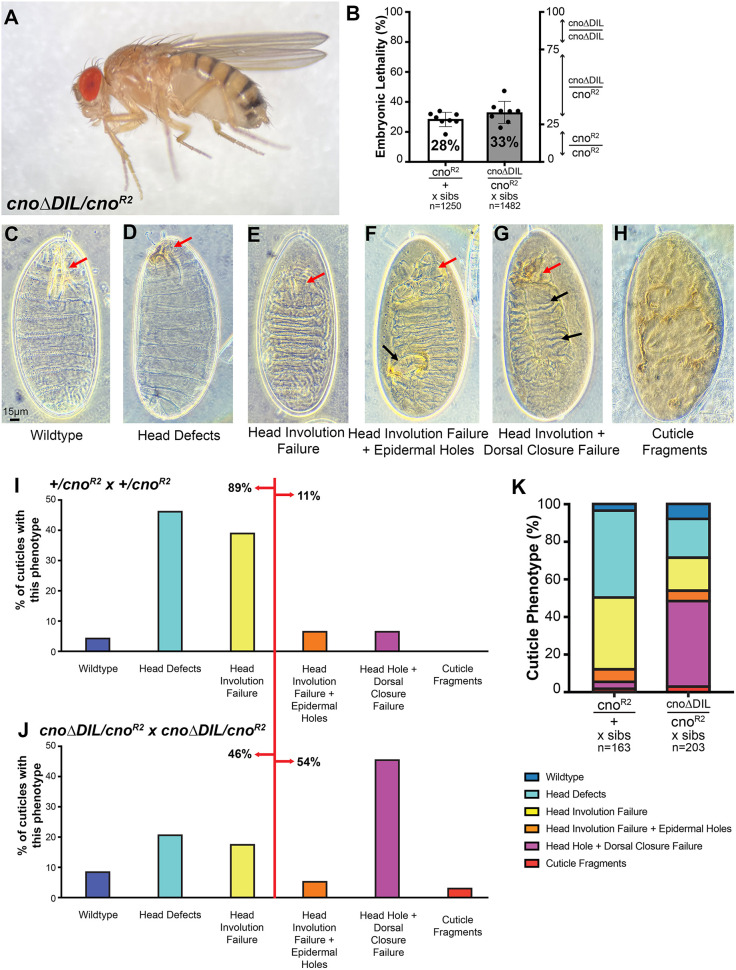
**CnoΔDIL is not required for viability, but a sensitized assay reveals roles in morphogenesis.** (A) *cno*Δ*DIL/cno^R2^* mutant (adults are ∼3 mm long). (B) *cno^R2^* is zygotically embryonic lethal, so 25% of progeny of heterozygous parents are expected to die as embryos. We observed 28% lethality. Embryonic lethality of the progeny of *cno*Δ*DIL/cno^R2^* parents was 33%, consistent with the possibility that some *cno*Δ*DIL/cno^R2^* embryos die. At least eight experiments were analyzed per genotype, with more than 1250 embryos analyzed in total. Each dot is the lethality observed in a single experiment. The mean and s.d. are indicated. When analyzed using Welch's unpaired two-tailed *t*-test using *n*=8 suggested the difference in lethality was not significant (*P*=0.15). (C–H) Potential cuticle phenotypes seen in different *cno* mutants, becoming more severe from left to right. Red arrows, head defects; black arrows, epidermal defects. (C) Wild type. Dorsal closure and head involution are completed correctly and the head skeleton is formed correctly (arrow). (D) Defects in the head skeleton (arrow). (E) Head involution failed, leading to a hole in the anterior end of the cuticle (arrow). (F) Head involution failed (red arrow) and there were holes in the dorsal or ventral cuticle (black arrow). (G) Head involution (red arrow) and dorsal closure failed, leaving the dorsal cuticle open (black arrows). (H) More severe defects in epidermal integrity. (I,K) In the *+/cno^R2^* cross, most progeny only have defects in head involution. (J,K) In the *cno*Δ*DIL/cno^R2^* cross, almost half of the progeny exhibit failure of both head involution and dorsal closure.

We first needed to determine whether *cno*Δ*DIL/cno^R2^* transheterozygotes are adult viable. We obtained both male and female *cno*Δ*DIL/cno^R2^* transheterozygotes at Mendelian ratios (33% of progeny from a cross of *cno*Δ*DIL/TM3*×*cno^R2^/TM3*) (TM3 is homozygous lethal; *n*=163 adults; Fig. 8A). We then used our sensitized test to determine whether *cno*Δ*DIL* provides wild-type Cno function. When we crossed *cno*Δ*DIL/cno^R2^* males and females, we observed slightly elevated lethality relative to the cross of *+/cno^R2^* males and females (Fig. 8B; 33%; *n*=1250 embryos versus 28% for *+/cno^R2^* parents; *n*=1482 embryos), but when analyzed by average lethality per experiment (*n*=8) this difference was not significant. This lethality was not as elevated as we had observed with *cno*Δ*PDZ* or *cno*Δ*FAB* crosses, in which most of the *cno*Δ*PDZ/cno^R2^* progeny or all of the *cno*Δ*PDZ/cno^R2^* progeny died as embryos (Perez-Vale et al., 2021).

We next examined whether having a maternal contribution of CnoΔDIL protein rather than wild-type Cno would enhance the embryonic morphogenesis phenotypes of the *cno^R2^* homozygous zygotic mutant progeny. We first assessed the larval cuticle, which provides a sensitive readout of many aspects of embryonic morphogenesis requiring cell adhesion and the connection to the cytoskeleton, ranging from germband extension and retraction to dorsal closure to head involution. The cuticle of wild-type embryos is intact, has a well-developed head skeleton, the result of successful head involution, and is closed dorsally (Fig. 8C). In most *cno^R2^* zygotic mutants derived from *+/cno^R2^* parents, germband extension, retraction and dorsal closure go to completion, such that the only defects in most embryos are in head involution, leading to a disrupted head skeleton (Fig. 8E,F; Gurley et al., 2023) – 89% of embryos were in these categories, whereas only 11% had more severe phenotypes (Fig. 8F–H; quantified in Fig. 8I,K). In contrast, in crosses of *cno*Δ*DIL/cno^R2^* parents, many more embryos exhibited defects in or complete failure of dorsal closure (Fig. 8F,G), with 54% of the embryos in more severe categories (quantified in Fig. 8I,K). This enhancement of phenotypic severity strongly suggests that CnoΔDIL does not provide fully wild-type Cno function.

**Fig. 9.**
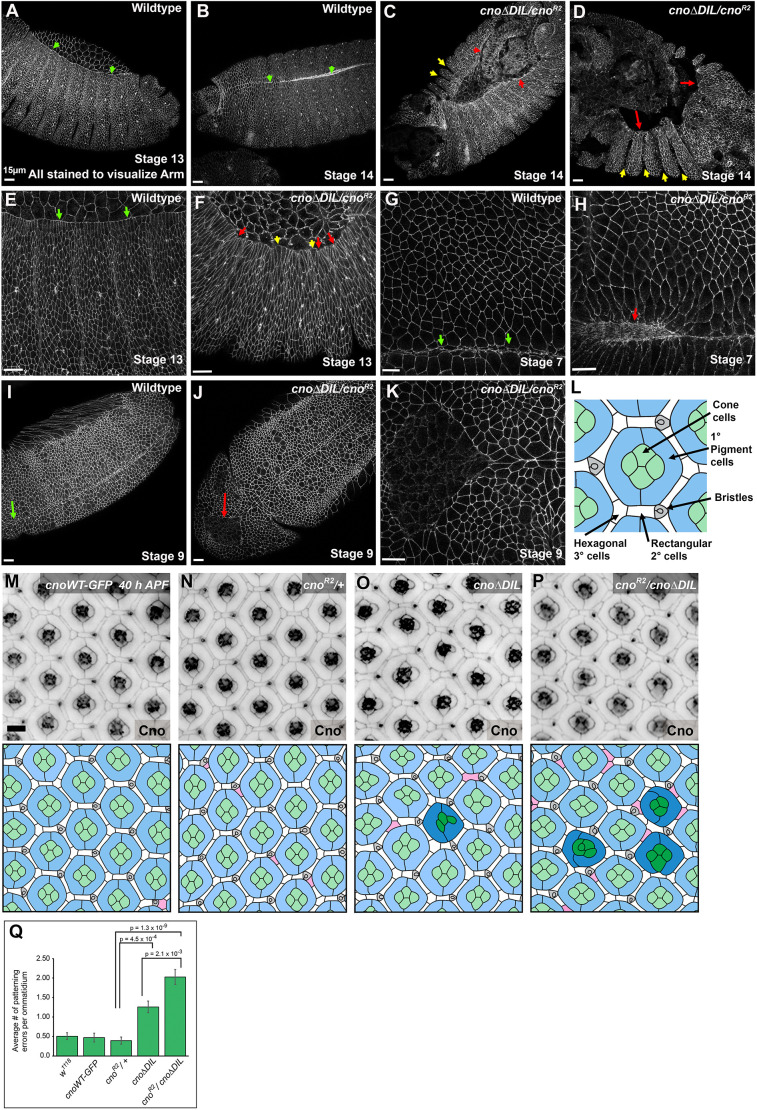
**CnoΔDIL does not provide fully wild-type function in embryonic morphogenesis or eye development.** (A–K) Embryos, anterior left, stages and antigens indicated. A–I, dorsal up; J,K, ventral view. Embryos labeled *cno*Δ*DIL/cno^R2^* are progeny of the cross of *cno*Δ*DIL/cno^R2^* parents – genotype was not determined. (A–F) Stages 13 and 14, mid-late dorsal closure. (A,B) During wild-type dorsal closure, as the amnioserosal cells constrict, the lateral epidermis extends dorsally (A, arrows) and zippers closed at the dorsal midline (B, arrows). (C,D) Dorsal closure failure in embryos from the *cno*Δ*DIL/cno^R2^* cross. The epidermal leading edge has detached from the amnioserosa (red arrows), which is undergoing apoptosis before closure is complete. Embryos also exhibit persistent deep segmental grooves (yellow arrows), a characteristic phenotype of strong *cno* mutants. (E) In wild type, leading edge cells extend dorsally, and cell width at the leading edge is relatively uniform (arrows). (F) In embryos from the *cno*Δ*DIL/cno^R2^* cross, some leading edge cells are hyperconstricted (red arrows) and some are splayed open (yellow arrows). (G,H) Stage 7 embryos. In wild type, the mesoderm is fully invaginated at the ventral midline (G, arrows), whereas in some embryos from the *cno*Δ*DIL/cno^R2^* cross there were mild defects in full mesoderm invagination (H). (I–K) Stage 9 embryos. In wild type, the ventral furrow is fully closed (I, arrow), whereas some embryos from the *cno*Δ*DIL/cno^R2^* cross have an open ventral furrow anteriorly (J, arrow; K). Quantitative information is provided by the cuticle results in Fig. 8. (L) Cartoon of the pupal eye ommatidium at 40 h APF. (M–P) Small regions of *cno-WT-GFP* (M), *cno^R2^/+* (N)*, cno*Δ*DIL-GFP* (O) and *cno^R2^/cno*Δ*DIL-GFP* (P) eyes dissected at 40 h APF. GFP-tagged Cno proteins were detected in M–P, and endogenous Cno in M. Tracings of each image are presented below, with the cone cells in green, 1° pigment cells in blue, bristle groups in grey, and lattice in white, as indicated in L. Patterning errors are indicated in darker shades of green or blue, and lattice cells that are incorrectly placed or shaped or in excess in the tissue are highlighted in pink. (Q) Patterning errors quantified as average number of patterning errors per ommatidium. Error bars are s.e.m. and significance was calculated using unpaired *t*-tests with Welch's correction (two-tailed *P-*value). Scale bars: 15 µm (A–K); 10 µm (M).

We also stained embryos to directly observe cell shape changes and morphogenetic movements. The strong maternal contribution of Cno only begins to diminish during dorsal closure, and thus embryos zygotically homozygous mutant for *cno^R2^* from heterozygous wild-type mothers only begin to exhibit morphogenetic defects at that stage (Choi et al., 2011; Gurley et al., 2023; Sawyer et al., 2009). Most *cno^R2^* zygotic mutant embryos complete dorsal closure and only exhibit defects in head involution (Gurley et al., 2023). Our cuticle data revealed that when the protein contributed maternally was CnoΔDIL, the *cno^R2^* zygotic mutant cuticle phenotype was enhanced, with many embryos exhibiting defects in dorsal closure (Fig. 8J). In wild-type embryos, dorsal closure is driven by amnioserosal apical constriction, leading edge cable contraction and leading-edge zipping at the canthi. Together these ensure that closure is complete before amnioserosal apoptosis (Fig. 9A,B). In contrast, in many embryonic progeny of crosses between *cno*Δ*DIL/cno^R2^* parents dorsal closure clearly failed, with separation of the amnioserosa and leading edge (Fig. 9C, red arrows) or complete failure to close before amnioserosal apoptosis (Fig. 9D, red arrows) – this was consistent with our cuticle data. We also observed other defects we previously observed in *cno* mutants (Manning et al., 2019), such as persistent deep segmental grooves (Fig. 9C,D, yellow arrows) and uneven cell shapes at the leading edge during dorsal closure (Fig. 9E versus 9F), with some cells hyperconstricted and others splayed open (Fig. 9F, red versus yellow arrows). In some embryos from the *cno*Δ*DIL/cno^R2^* cross, we observed one additional earlier defect that was not previously seen in *cno* zygotic mutants but is present in maternal and zygotic *cno* mutants – defects in completion of mesoderm invagination. We observed these defects in 36% of embryos from the *cno*Δ*DIL/cno^R2^* cross. Defects included both mild defects along the ventral midline in which some mesoderm was exposed (Fig. 9G versus H; 9/39 embryos scored) and more severe defects in which the ventral furrow remained open at the anterior or posterior end (Fig. 9I versus J,K; 5/39 embryos). The frequency of defects was substantially higher than that observed in the *+/cno^R2^* cross (6%= 3/51 had mild defects, 0/51 were anterior open) or in wild-type embryos (6%; 2/34 had mild defects, 0/34 were anterior open). Taken together, these data reveal that CnoΔDIL does not retain fully wild-type function.

### The DIL domain of Cno is required for its role in patterning the developing eye

Cell shape change and tissue rearrangement are not confined to embryonic development. The developing eye provides another outstanding place to explore how AJ–cytoskeletal connections shape tissue morphogenesis. The ∼750 ommatidia of the mature eye emerge during pupal development from a neuroepithelium that becomes precisely patterned so that, when examined 40 h after pupal development has begun, each nascent ommatidium has a stereotyped arrangement of epithelial cell types. Each cell type is easily identified due to its characteristic shape (Johnson, 2021). Clusters of four cone cells sit at the center of each ommatidium, surrounded by two primary (1°) pigment cells, and each of the ∼750 ommatidia are separated by a neat lattice of rectangular secondary (2°) and hexagonal tertiary (3°) pigment cells, as well as bristle precursors (Fig. 9L,M). Cno is important for the proper development of ommatidial architecture – weak alleles alter the stereotyped arrangement of cells (Gaengel and Mlodzik, 2003; Matsuo et al., 1997, 1999), whereas complete loss of function dramatically disrupts epithelial architecture (Walther et al., 2018).

In our previous work, we found that although *cno*Δ*PDZ* and *cno*Δ*FAB* mutants had only subtle defects in embryonic development, they had penetrant defects in cell arrangements in the developing pupal eye (Perez-Vale et al., 2021), suggesting that this tissue provided a more sensitive place to examine protein function. We thus examined pupal eye development in *cno*Δ*DIL* mutants, using wild-type flies (mutant for the *white* gene), flies carrying a GFP-tagged wild-type *cno* gene (Cno-WTGFP), and flies heterozygous mutant for the *cno*-null allele *cno^R2^* as controls. *cno*Δ*DIL* homozygous mutant pupae had an elevated number of defective ommatidia with incorrect cell arrangements (Fig. 9O,Q; Table S1). The cone cells were occasionally mis-configured, ommatidia were sometimes observed with fewer than four cone cells, and the shapes of 1° cells were less ordered (Fig. 9O). The lattice was also less precise, with lattice cells occasionally misplaced or incorrectly shaped. Quantifying total defects, using our previously developed scoring scheme (Johnson and Cagan, 2009), verified the increase in defect frequency (Fig. 9Q) – the defect frequency in *cno*Δ*DIL* homozygotes was roughly comparable to the defect frequency we saw in *cno*Δ*FAB* mutants and somewhat less severe than we observed in *cno*Δ*PDZ* (Perez-Vale et al., 2021). Patterning errors were enhanced in flies in which *cno*Δ*DIL* was heterozygous with *cno^R2^*, thus reducing protein levels (Fig. 9P versus 9N,O; Fig. 9Q), with errors in cone-cell and 1° cell configuration consistent with altered cell adhesion during the earlier morphogenesis of these cells. Thus, the DIL domain is important for the role of Cno in eye development.

## DISCUSSION

The dramatic events of embryonic morphogenesis depend on establishing and maintaining robust yet dynamic connections between cell–cell AJs and the actomyosin cytoskeleton. We seek to define the molecular mechanisms by which these connections are made via a network of interconnected proteins. Cno and its mammalian homolog afadin are central players in this network. In their absence, the complex events of gastrulation and other morphogenetic movement fail, as AJ–cytoskeletal connections are disrupted at places where force is exerted on junctions. Cno provides a superb entry point for defining molecular mechanisms as it, like many AJ proteins, is a complex multidomain protein, with its different domains allowing multivalent connections to diverse other proteins in the network. We are systematically exploring the role of the many folded protein domains in Cno for its biochemical and cell biological functions.

### The DIL domain of Cno can dimerize and has a prominent conserved groove on its surface

The DIL domain is the largest of the conserved folded domains in Cno and afadin, but we had little information about its biochemical or biological functions. The DIL domain in MyoV, the only other protein family in which it occurs, provided speculative possibilities. In MyoV, the DIL domain serves two major functions – it acts as an interface for multiple intramolecular and intermolecular interactions, and it can dimerize the protein.

The new tools available from AlphaFold (Jumper et al., 2021; Varadi et al., 2022) provided an opportunity to compare the predicted structure of the DIL domains of Cno and afadin with that of MyoV. The predicted structures of the Cno and afadin DIL domains are strikingly similar to those of different MyoV family members, with 15 α-helices forming an elongated domain, as in the MyoV DIL domain. Differences between MyoV and Cno/afadin are largely confined to a subset of the interhelical loops and the C-terminal 15th α-helix. Cno and afadin are even more similar to one another in predicted structure, with only a few minor differences in interhelical loops. Examining sequence conservation between *Drosophila* Cno and mammalian afadin provided additional insights. Intriguingly, the regions that mediate the many intramolecular and intermolecular interactions of MyoV are not well conserved between Cno and afadin. This includes the region of MyoV that binds Rab GTPases, which was of particular interest because of the known regulation of Cno and afadin by the small GTPase Rap1. Instead, although Cno and afadin only share 48% overall identity, there is very strong conservation of a groove on the surface of the DIL domain consisting of residues from α6, α7, α9 and α10 that are distinct from those found in MyoV. We suspect this groove serves as a protein interaction site, and it will be of interest to determine the nature of the potential intramolecular or intermolecular interactions occurring here. One resource will be proteins that were identified as afadin neighbors using proximity labeling approaches like BioID (Baskaran et al., 2021; Goudreault et al., 2022). It will also be of interest to explore potential intramolecular interactions, for example with predicted helical regions in the Cno and afadin IDRs (Gurley et al., 2023).

We now have in hand structures or predicted structures of all five folded protein domains that make up the N-terminal half of the Cno/afadin proteins. What we lack is any information about how they interact with one another. We do know that the N- and C-termini of the DIL domain are in close proximity on one end of the elongated predicted structure, and that the linker between the DIL domain and the PDZ domain that immediately follows is quite short (6–10 amino acids). Given that the PDZ domain links Cno to the C-terminal tails of transmembrane adhesion receptors like E-cadherin and nectins, that will place the DIL domain relatively close to the plasma membrane, and that end of the DIL domain is positively charged, which might complement the negatively charged membrane lipids. We are keenly interested in using either structural or computational approaches to see whether these five domains and their binding partner, Rap1, occur in one or several quaternary conformations that might regulate activity. However, the fact that one can delete either the DIL domain or the PDZ domain without drastically disrupting Cno function calls into question the importance of hypothetical structures such as these, unless they can accommodate a major structural change.

One property conferred on MyoV by its DIL domain is the ability to dimerize. We wondered whether the DIL domain of Cno shared this property. Using SEC-MALS, we found that the purified DIL domain of Cno can dimerize *in vitro*. We observed both monomer and dimer populations, suggesting a relatively weak interaction. However, in AJs, clustering might increase local concentrations, favoring dimerization. We also observed dimerization of a longer protein encompassing the FHA, DIL and PDZ domains, though at a lower frequency. This property might contribute to the ability of both afadin and Cno to dimerize and/or oligomerize *in vivo* (Bonello et al., 2018; Mandai et al., 1997), but it will be important in the future to explore potential roles for the DIL domain in the oligomerization we and others have observed *in vivo*. We suspect interactions in the AJ complex are multivalent, including both direct and indirect interactions among the proteins in the network, and thus Cno and afadin might self-associate via multiple means.

### Cno recruitment to AJs involves multivalent interactions, as deleting individual protein domains does not prevent it

Our CRISPR-based genetic platform allows us to replace the *cno* coding sequence at the locus with a version that cleanly lacked the DIL domain. Loss of the DIL domain did not alter its recruitment to AJs from their initial establishment to the end of morphogenesis. It also did not alter enrichment of Cno protein at the AJs, which are under elevated tension. In this way, it resembled two of the mutants we examined earlier, *cno*Δ*PDZ* and *cno*Δ*FAB* (Perez-Vale et al., 2021). Although the tandem RA domain cassette and its Rap1 binding partner are important for initial Cno localization to AJs as they first assemble, they become dispensable for AJ localization after gastrulation onset (Perez-Vale et al., 2023, 2021). Thus, four of the five folded domains present in Cno, along with its conserved C-terminal FAB, are each singly non-essential for AJ recruitment, despite their conservation from *Drosophila* to mammals, over ∼600 million years of evolutionary time. These data reinforce the remarkable multivalent nature of AJ assembly, with multiple interactions appearing to be sufficient for Cno recruitment to AJs. In future studies, it will be important to test the FHA domain and the remainder of the IDR – perhaps one of those might be essential for AJ localization. Moving on, mutating multiple domains simultaneously might help reveal potential redundancy.

### The DIL domain of Cno is not essential for viability but plays a supporting role in Cno function in morphogenesis

The conservation of the DIL domain in all animal family members led us to suspect it would play an important role in Cno/afadin function *in vivo*. However, to our surprise, *cno*Δ*DIL* mutants are viable and exhibit normal or near normal fertility. Closer examination of *cno*Δ*DIL* mutants during morphogenesis verified normal function during cellularization, germband elongation and dorsal closure, times at which AJs need to dynamically respond to tension as cells change shape. It was only when we reduced CnoΔDIL levels, using our sensitized assay, in which we examined the progeny of *cno*Δ*DIL/cno^R2^* parents, that its importance in maintaining robustness became apparent. CnoΔDIL could not provide full function in embryos lacking maternal and zygotic wild-type Cno, and thus the morphogenesis defects of *cno* zygotic-null mutants were substantially enhanced, with increased failure of dorsal closure and failure to fully internalize the mesoderm.

How can a protein domain that has been maintained through 600 million years of evolutionary divergence (Gurley et al., 2023) be dispensable for embryonic morphogenesis? Together with our earlier work on *cno*Δ*PDZ* and *cno*Δ*FAB* (Perez-Vale et al., 2021), these data emphasize the power of natural selection to maintain protein structure even when mutants appear wild-type or near wild-type in our lab-based assays. In the case of *cno*Δ*FAB* mutants, the 25% embryonic lethality would provide ample scope for selection. We think it is likely that *cno*Δ*PDZ* and *cno*Δ*DIL* mutants have defects in viability and morphogenesis that are within the noise of our phenotypic analysis, and that the activity that these domains confer was sufficient to maintain Cno/afadin protein structure and to maintain substantial sequence conservation. Furthermore, *cno*Δ*DIL* mutants share defects in the precision of eye development with cnoΔ*PDZ* and *cno*Δ*FAB* – these and potential similar issues like this in other internal and/or postembryonic tissues are also likely to be sufficient to empower selection. We imagine similar constraints explain the maintenance of genes like *sidekick* and *vinculin* in which animals completely lacking these proteins are viable and fertile – *sidekick* mutants have defects in ommatidial development similar to those we observed here (Letizia et al., 2019). Together, these emphasize the need for robustness in AJ–cytoskeletal connections during multiple embryonic and postembryonic events, and the need to experimentally map and characterize the multivalent interactions that underlie the network.

## MATERIALS AND METHODS

### Cloning and purification of Cno DIL domain construct

The Cno DIL domain sequence [amino acids (aa) 613–1006] was cloned into plasmid pET28 (Millipore Sigma, Burlington, MA, USA) using PCR and primers with engineered NheI and EcoRI restriction sites. The Cno FHA-DIL-PDZ coding region (aa 372–1110) was cloned into a modified pET28 plasmid (Millipore Sigma, Burlington, MA) with an engineered an N-terminal PreScission (Cytiva, Marlborough, MA) protease-cleavable hexa-histidine tag. Cloning used PCR and primers with engineered NheI and EcoRI restriction sites. Plasmids containing the respective constructs were transformed into BL21 (DE3) pLysS *E. coli* cells (Thermo Fisher Scientific, Waltham, MA ) and grown in 8 l LB medium (Thermo Fisher Scientific) with 20 mg/l kanamycin (Thermo Fisher Scientific) at 37°C. After reaching an optical density of 0.8 at 600 nm, protein expression was induced with 100 μM IPTG (UBPBio, Aurora, CO) for 16 h at 20°C. Cells were harvested by centrifugation at 2000 ***g*** at 4°C, then were resuspended in 200 ml buffer A (25 mM Tris-HCl pH 8.5, 300 mM NaCl, 0.1% β-mercaptoethanol, 10 mM imidazole; Thermo Fisher Scientific) supplemented with 5 µg/ml DNase (Roche Diagnostics, Mannheim, Germany), 10 µg/ml lysozyme (Thermo Fisher Scientific) and 0.5 mM PMSF (BIOSYNTH International, Itasca, IL). Cells were lysed by sonication. Lysate was clarified by centrifugation at 23,000 ***g*** for 45 min at 4°C. Supernatant was loaded onto a nickel-nitriloacetic acid (Ni^2+^-NTA) column (Qiagen, Hilden, Germany), washed with 500 ml buffer A, and eluted in 75 ml buffer B (buffer A plus 290 mM imidazole). To the eluate containing Cno DIL, 25 μl thrombin (6 mg/ml; Prolytix, Essex Junction, VT, USA) and CaCl_2_ (1 mM final concentration) was added. To the eluate containing Cno FHA-DIL-PDZ, 50 μl PreScission protease (5 mg/ml; Cytiva, Marlborough, MA, USA) was added. Cleavage of the His_6_ tag via proteolysis occurred overnight at 4°C. Digested Cno constructs were dialyzed overnight against 25 mM Tris-HCl pH 8.5, 300 mM NaCl and 0. 1% β-mercaptoethanol (all from Thermo Fisher Scientific), and filtered over Ni^2+^-NTA resin. The Cno DIL construct was also filtered over benzamidine Sepharose (Cytiva). Proteins were exchanged into 25 mM Tris-HCl pH 8.5, 300 mM NaCl, and 0.1% β-mercaptoethanol, concentrated in a 30,000 MWCO Millipore concentrator to 85 mg/ml (Cno DIL) and 65 mg/ml (Cno FHA-DIL-PDZ), and flash frozen in liquid nitrogen for storage at −80°C.

### SECMALS data collection

Purified and concentrated Cno DIL domain protein was thawed at room temperature and diluted to 5.0 mg/ml in 300 mM NaCl, 25 mM Tris-HCl pH 8.5, 1 mM MgCl_2_, 0.1% β-mercaptoethanol and 0.2 g/l sodium azide to a total volume of 120 μl. 100 μl of diluted protein was injected onto a Superdex 200 column with a flow rate of 0.5 ml/min. The protein passed through a Wyatt Optilab rES refractometer followed by a DAWN HELEOS II light-scattering instrument. Refractive index and light-scattering data were processed using the Astra software program (Waters Corporation, Milford, MA) and were used to determine the molar mass of the protein. The SEC-MALS data shown is representative of experiments conducted in duplicate using a biological replicate.

### Structure prediction and structure analysis

*Drosophila* Cno and rat afadin structure prediction models were obtained from the AlphaFold server (Jumper et al., 2021; Varadi et al., 2022). The MyoVb DIL domain was obtained from PDB 4J5M (Nascimento et al., 2013). Coordinates of MyoV-binding proteins in complex with the MyoV DIL domain are from PDB 4KP3 (RILPL2; Wei et al., 2013), 4LX0 (Rab11; Pylypenko et al., 2013), 4KP3 (melanophilin; Wei et al., 2013), 5JCY (Spire2; Pylypenko et al., 2016), and 6KU0 (MICAL1; Niu et al., 2020). Coordinates of the full-length MyoVa structure are from PDB 7YV9 (Niu et al., 2022). Structures were aligned using the align command in PyMOL (Schrodinger). Protein sequence alignments were generated using Clustal Omega (Madeira et al., 2022), and adjusted manually based on output from the PyMOL structural alignments.

### Fly work

We used *yellow white* flies as our control, and we refer to them in the text as wild type. All the experiments were performed at 25°C.

### Generating the mutant rescue construct and its ΦC31-mediated integration into the *cno*ΔΔ allele attP site

The *cnoWT-GFP* rescue construct (Perez-Vale et al., 2021) was used to generate a new construct lacking the DIL domain. To remove this domain, we designed an in-frame deletion of aa 613–993 of *Drosophila* Cno. We created this deletion using a Q5 Site-Directed Mutagenesis kit (New England Biolabs, Cat. No: E0554S) with subsequent sequence verification. The vector carrying the *cno*Δ*DIL* gene, pGE-attB-GMR, also carries a *w*^+^ selectable marker next to the *cno* coding sequence, and both are flanked by attR and attL sites allowing site-specific integration into the attP site at the *cno*ΔΔ locus (Perez-Vale et al., 2021)*.* Injection of the *cno*Δ*DIL*–GFP rescue construct was carried out by BestGene (Chino Hills, CA, USA); this DNA was injected into *PhiC31/int^DM Vas^; cno*ΔΔ embryos. F1 offspring were screened for the presence of the *w*^+^ marker and outcrossed to *w; TM6B, Tb/TM3, Sb* to generate a balanced stock over TM3. We verified the integration of *cno*Δ*DIL-GFP* by both PCR amplification and sequencing and by immunoblotting. To remove potential other mutations from the *cno*Δ*DIL-GFP* chromosome we outcrossed the stock to a *y w* stock with a wild-type third chromosome for multiple generations, selecting for the linked *w*^+^ marker in each generation. This allowed us to homozygose *cno*Δ*DIL-GFP.* The *cno*Δ*DIL-GFP* stock will be made available via the Bloomington *Drosophila* Stock Center.

### Molecular characterization of engineered *cno* allele

The following primers were used for PCR amplification of the sequence between the intron upstream of construct insertion and the exon after RA1: forward (F.1), 5′-ACCGTCACAAACCAACCAGA-3′; reverse (R.1), 5′-AACACCATTTCCCAAGCCCA-3′. The RA1 domain is encoded by two exons which, in the wild-type coding sequence (CDS), are separated by an intron sequence. Because ΔDIL mutants lack wild-type introns after the 5′ UTR, the amplicon produced from ΔDIL mutants is expected to be smaller in size compared to that of wild type. For PCR amplification of the sequence between the end of FHA and the first PDZ exon, the following primers were used: forward (F.2), 5′-GTGGTAATGTTCGGACGGGT-3′; reverse (R.2), 5′-CCAGGCACCACACTCTTGAT-3′. In the wild-type CDS, there exist several introns between the FHA and the beginning of the PDZ domain. Again, because ΔDIL mutants lack introns after the 5′ UTR, the amplicon produced from ΔDIL mutants is expected be smaller in size compared to that of wild type. Finally, for PCR amplification of the sequence between the end of FHA and the intron after the first DIL exon, the following primers were used: forward (F.3), 5′-GTGGTAATGTTCGGACGGGT-3′; reverse (R.3), 5′-AATGGCGGCTGCTTTCAT-3′. In this pairing, because the reverse primer targets an intron sequence that is non-existent in ΔDIL mutants, an amplicon is only produced for wild type. Predicted sizes are in Table S2.

### Embryo fixation and immunofluorescence

Eggs were collected in cups at 25°C on apple juice agar plates with yeast paste. Embryos were dechorionated in 50% bleach, washed three times in 0.03% Triton X-100 with 68 mM NaCl, and then fixed in 95°C Triton salt solution (0. 03% Triton X-100 with 68 mM NaCl, 8 mM EGTA) for 10 s. We then added ice-cold Triton salt solution and transfer to ice for fast cooling for at least 30 min. We devitellinized the embryos by vigorous shaking in 1:1 heptane:methanol solution. The embryos were then washed three times with 95% methanol and 5% EGTA. After this step embryos were sometimes stored in 95% methanol and 5% EGTA at −20°C overnight before staining. Before staining, the embryos were washed three times with 5% normal goat serum (NGS; Thermo Fisher Scientific) and 0.1% saponin in phosphate-buffered saline (PBS) (PBSS-NGS). We then blocked in 1% NGS in PBSS-NGS for 1 h, and embryos were incubated in primary antibodies overnight at 4°C or 2–3 h at room temperature. Once incubation finished, we washed three times with PBSS-NGS and incubated embryos in secondary antibodies overnight at 4°C or 2–3 h at room temperature. Both primary and secondary antibodies were diluted in 1% bovine serum albumin and 0.1% saponin in PBS, and the dilutions used are listed in Table S3. After the secondary antibody incubation, we washed three times with PBSS-NGS and stored embryos in 50% glycerol until mounted on glass slides using a homemade Gelvatol solution (recipe from the University of Pittsburg's Center for Biological Imaging).

### Image acquisition and analysis

Fixed embryos were imaged on a confocal laser-scanning microscope (LSM 880; 40×/NA 1.3 Plan-Apochromat oil objective; Carl Zeiss, Jena Germany). Images were processed and maximum intensity projections were generated using ZEN 2009 software. We used Photoshop (Adobe, San Jose, CA) to adjust input levels and brightness and contrast. Analysis of apical-basal positioning on maximum intensity projections (MIPs) was executed as previously described (Choi et al., 2013). Briefly, using Zen 2009 software, the *z*-stacks were cropped to select a region of interest (ROI) on the *xy*-axis of 250×250 pixels for stacks collected using a digital zoom of 2 or 200×200 pixels for stacks with a 1.6 digital zoom. Using the Zen software the *z*-stacks ROIs dimensions were modified from *yzx* to *xyz* along the *y*-axis from which MIPs were generated.

### Cno SAJ and TCJ enrichment analysis

Data for analyzing spot adherens junction (SAJ) and TCJ enrichment was obtained from *z*-stacks taken through the embryo using a digital zoom of 1.6 or 2 and a step size of 0.3 µm. First, the total length of the cells was defined by determining slice position of the apical (top) and basal (bottom) of the cells in the stack. For embryos in mid-late stage 5, the SAJs were determined to be at 21.82% of the total length and the TCJ enrichment was assessed at 33.33% more basal to the SAJs. For embryos in late stage 5, the SAJs correspond to 21.82% of the total length and the TCJ enrichment was assessed at 50% more basal to the SAJs.

The Cno TCJ intensity ratio was measured from MIPs of a 1.2–2.4 µm of the apical AJ region of embryos from stage 7. The MIPs were generated from *z*-stacks taken through the embryo using a digital zoom of 1.6 or 2 and a step size of 0.3 µm. ImageJ software was used to identify the apical AJ region from which *z*-stack MIPs were generated. The mean intensity of Cno was measured using FIJI software (National Institutes of Health, Bethesda, MD, USA) by creating lines using the line tool (line width of 5 pixels) along the bicellular junctions, avoiding TCJs or multicellular junctions, and next creating short lines at TCJ or multicellular junctions at 300% zoom. For each TCJ or four-way junction, the three or four bicellular junctions in contact with that junction were measured to obtain the mean intensity. For each junction, a short line was drawn in the cytoplasm to standardize pixel intensity for the image by subtracting cytoplasmic background from the junctional intensity. A total of ten cells were quantified per embryo and a total of four embryos were assessed from three experiments. The average bicellular junction intensity per cell was calculated. The Cno TCJ ratio was calculated by dividing the mean intensity of the TCJ by the average of the bicellular junctions. Box-and-whisker graphs were made using GraphPad: the box shows the 25^th^–75th percentile, the whiskers are 5th–95th percentiles, the horizontal line is the median and the plus sign (+) is the mean. Data statistical analysis was done using GraphPad. Statistical significance was calculated by Welch's unpaired *t*-test or Brown Forsythe and Welch ANOVA test.

### Planar polarity quantification

The planar polarity of Cno was measured from MIP of a 2.4 µm region of the apical AJs of embryos from stage 7 to early stage 8. The MIPs were generated from *z*-stacks taken through the embryo using a digital zoom of 1.6 or 2 and a step size of 0.3 µm. Using FIJI software, the 2.4 µm region was identified from which MIPs were generated. The mean intensity was measured using FIJI software by creating lines (line width of 5 pixels) at 300% zoom at bicellular borders, without including the TCJ or multicellular junctions. AP and DV borders were selected manually, choosing cells with aligned AP borders where planar polarity is most apparent. The background intensity was measured by drawing a short line in the cytoplasm of all cells measured. The background pixel intensity was subtracted from AP and DV border intensities. A total of four embryos were assessed from at least four experiments. Cno was normalized to DV borders producing an AP/DV ratio. Box-and-whisker graphs were made using GraphPad. The box shows the 25th–75th percentile, the whiskers show 5th–95th percentiles, the horizontal line shows the median and the plus sign (+) is the mean. Data statistical analysis was done using GraphPad. Statistical significance was calculated using a one-way ANOVA with Sidak's multiple comparisons post test.

### Cuticle preparation and analysis

We prepared embryonic cuticles according to (Wieschaus and Nüsslein-Volhard, 1986). Embryos were collected on apple juice agar plates with yeast, aligned on a fresh apple juice agar plate without yeast and incubated at 25°C for 48 h to allow embryos to develop fully and viable embryos to hatch. All unhatched embryos were collected in 0.1% Triton X-100 and dechorionated in 50% bleach for 5 min. They were then washed three times with 0. 1% Triton X-100 and transferred to glass slides, where all the liquid was removed, mounted in 1:1 Hoyer's medium:lactic acid, and incubated at 60°C for 24–48 h. They were then stored at room temperature. Images were taken using a Nikon Labophot with a 10× Phase 2 lens, and captured on an iPhone, and placed into categories based on morphological criteria.

### Western blotting

Table S3 contains the antibodies and dilutions used for these experiments. Protein levels expression of Cno, Pyd and Arm were determined by immunoblotting embryos collected in the 1–4 h and 12–15 h windows. The lysates were generated as in Manning et al. (2019). Briefly, embryos were dechorionated for 5 min in 50% bleach. After washing three times with 0.1% Triton X-100, lysis buffer [1% NP-40, 0.5% Na deoxycholate, 0.1% SDS, 50 mM Tris-HCl pH 8.0, 300 mM NaCl, 1.0 mM DTT, Halt™ Protease and Phosphatase Inhibitor Cocktail (Thermo Fisher Scientific, #78442) (100×), and 1 mM EDTA] was added and the embryos were placed on ice. Embryos were ground in a microcentrifuge tube using a pestle, lysate was centrifugated at 16,361 ***g*** for 15 min at 4°C, and protein concentration was determined using Bio-Rad Protein Assay Dye. The lysates were resolved using 7% SDS-PAGE and transferred onto nitrocellulose membranes with a pore size of 0.2 μm. The membranes were blocked in 10% bovine serum albumin (BSA) diluted in Tris-buffered saline with 0.1% Tween-20 (TBST) for 1 h at room temperature. For primary and secondary staining, antibodies were diluted in 5% BSA with TBST. Incubation was performed either for 2 h at room temperature or overnight at 4°C for the primary antibody, and a 45-min incubation at room temperature was performed for the secondary antibody. The membranes were developed using the Odyssey CLx infrared system (LI-COR Biosciences). Analysis of band densitometry was calculated using Empiria Studio^®^ Software.

### Pupal eye dissection, immunofluorescence, and analysis

Wild-type and mutant stocks were maintained on nutrient-rich *Drosophila* media at 25°C. Pre-pupae were selected and maintained in humidified chambers until dissection at 40 h after puparium formation (APF) (DeAngelis and Johnson, 2019). Rabbit anti-Cno (1:500) and chicken anti-GFP (1:8000, Abcam #13970) followed by Alexa-Fluor-488-conjugated secondary antibodies (Jackson ImmunoResearch #711-545-152 or #703-545-155) were used to detect Cno, CnoWT-GFP and *cno*Δ*DIL-GFP*, and retinas imaged with a Leica DM5500 B fluorescence microscope. We performed dissections in triplicate, with 5–10 pupae of each genotype dissected each time. Patterning errors were scored in retinas from one representative dissection of three carried out in triplicate, as previously described (Johnson and Cagan, 2009). Analyses spanned 9–15 eyes for each genotype, with 110 data points (ommatidia) per genotype. Significance was assessed using unpaired *t*-tests with Welch’s correction (two-tailed *P*-value). Image files were processed for publication using Adobe Photoshop.

## Supplementary Material



10.1242/joces.261734_sup1 Supplementary information
