# Supplementary material for: The Dilute domain in Canoe is not essential for linking cell junctions to the cytoskeleton but supports morphogenesis robustness
Source: J Cell Sci. 2024 Mar 21;137(6):jcs261734. doi: 10.1242/jcs.261734 (PMC11006394; doi:10.1242/jcs.261734)
Supplement: Supplementary information [file joces-137-261734-s1.pdf]

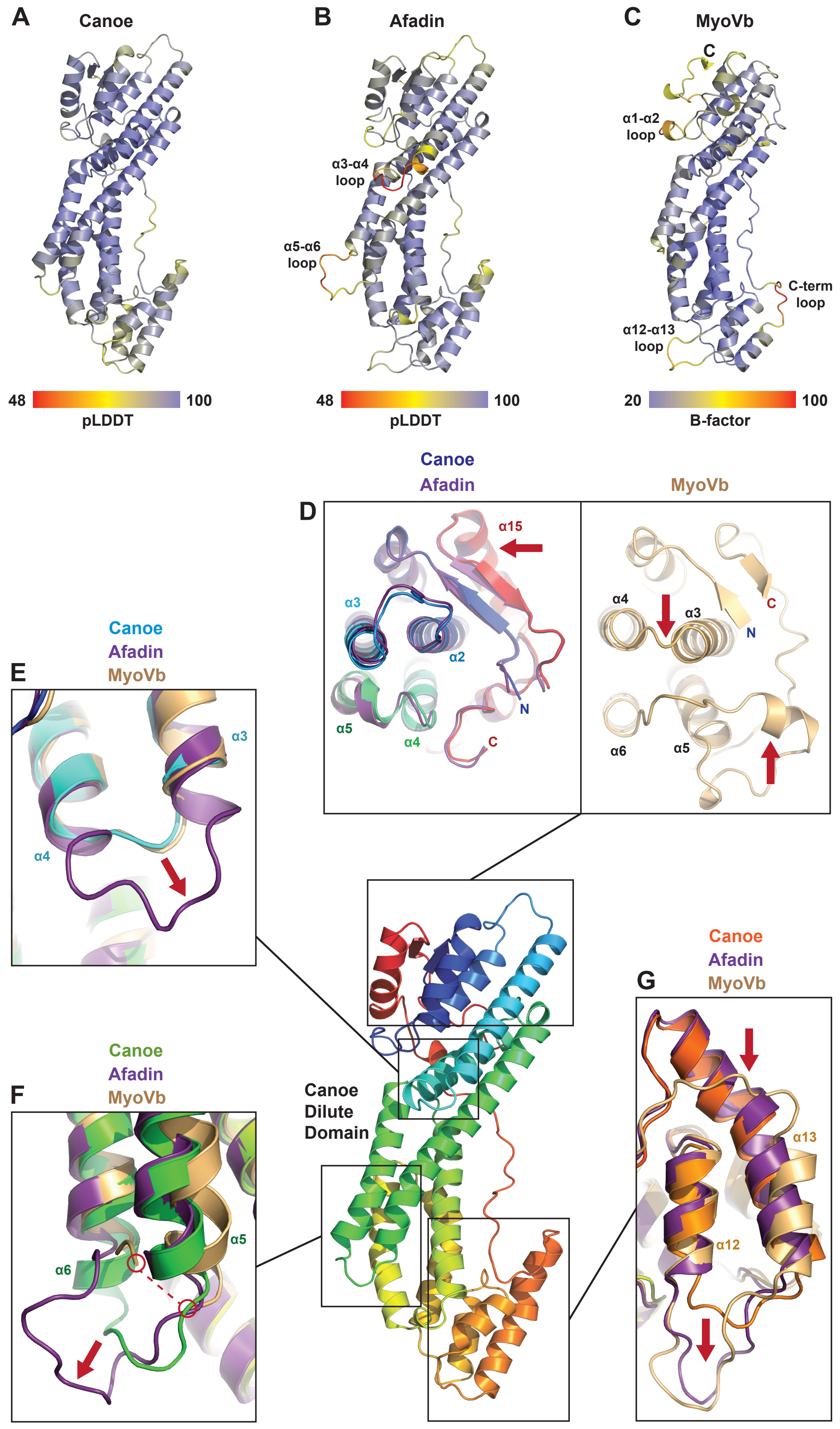

Journal of Cell Science • Supplementary information

Fig. S1. The predicted DIL domain structures of Cno and Afadin are structurally similar to the MyoVb DIL domain but have components that structurally diverge. (A,B) AlphaFold models of the Cno (A) and Afadin (B) DIL domains with pLDDT structure prediction confidence values mapped on the models. (C) The experimentally determined crystal structure of the MyoVb DIL domain with B-factors mapped on the model (PDB 4J5M (Nascimento et al., 2013)). (D-G) Center: Cno DIL domain AlphaFold structure prediction, shown in cartoon format, colored as in Figure 1C. Regions boxed are shown in more detail in D-G, and include structural alignments with the predicted structure of Afadin, and the experimentally determined structure of MyoVb from PDB 4J5M (Nascimento et al., 2013)). (D) Zoom view of the top of the DIL domains (rotated 90° about the x-axis relative to the center image) after structural alignment of the Cno and Afadin (shown at left) and MyoVb (shown at right) DIL domains. The Cno and Afadin predicted models have high structural homology over this region, while MyoVb has a unique helical insert (lower red arrow in right panel) between its α5 and α6 helices that occupies space which in the Cno/Afadin DIL domain models are predicted to be occupied by each domain's C-terminal tail. Cno and Afadin have a distinct predicted orientation of the α2-α3 loop that deviates from the corresponding MyoVb α3-α4 loop (right panel, upper red arrow). Cno and Afadin also have a predicted C-terminal helix, α15, that is not present in the MyoVb structure (red arrow, left panel). (E) Zoom view showing structural differences in the positioning and length of the predicted Cno and Afadin α3-α4 loop (red arrow), showing that the Cno α3-α4 loop is positioned similar to the corresponding loop in MyoVb. (F) Zoom view showing the variation in the positioning of the predicted α5-α6 loop of Cno and Afadin, which is not ordered in the MyoVb structure (MyoVb disordered loop indicated by a bridging red-dotted line). (G) Zoom view of the predicted Cno and Afadin DIL domain α12 - α13 region, showing variation in the positioning and length of the α12-α13 loop (lower red arrow), and the different length of the α13 helix (upper red arrow) which is extended in both the Cno and Afadin models, but is relatively shorter in the MyoVb structure.

Journal of Cell Science • Supplementary information

Table S1. Analyses of patterning defects in retinas

	Patterning errors per data point												Total errors per data point (ommatidium + surrounding lattice)				
	cone cell defects (1.)		1º cell defects (2.)		ommatidial misorientations (3.)		bristle defects (4.)		3º cell defects (5.)		errors in lattice cell number (6.)						
**Genotype**	**Mean**	**Std Dev**	**Mean**	**Std Dev**	**Mean**	**Std Dev**	**Mean**	**Std Dev**	**Mean**	**Std Dev**	**Mean**	**Std Dev**	**Mean**	**Std Dev**	**Std Error**	**comparison with *w*[1118] (p-value)**	**comparison with *cno-ΔDIL-GFP* (p-value)**
w[1118]	**0.00**	0.00	**0.01**	0.18	**0.00**	0.00	**0.28**	0.45	**0.10**	0.30	**12.13**	0.49	**0.51**	0.94	0.09		
w[1118]; *cno*[R2] / +	**0.00**	0.00	**0.01**	0.10	**0.00**	0.00	**0.05**	0.21	**0.06**	0.28	**12.23**	0.56	**0.40**	0.80	0.09	**0.0485**	
w[1118]; *cno-wt-GFP*	**0.00**	0.00	**0.01**	0.10	**0.01**	0.10	**0.16**	0.40	**0.16**	0.50	**11.93**	0.48	**0.47**	1.19	0.11	**0.3771**	
w[1118]; *cno-ΔDIL-GFP*	**0.16**	0.48	**0.20**	0.66	**0.00**	0.00	**0.14**	0.34	**0.44**	0.67	**11.81**	0.61	**1.26**	1.57	0.15	**4.503×10^{-4}**	
w[1118]; *cno-ΔDIL-GFP / cno*[R2]	**0.33**	0.49	**0.06**	0.31	**0.04**	0.19	**0.40**	0.59	**0.46**	0.65	**11.44**	0.96	**2.03**	2.05	0.19	**1.309×10^{-9}**	**2.105×10^{-3}**

The following defects were scored in 110 data points per genotype:
1. errors in cone cell number and arrangement
2. incorrect number, relative size and junctional integrity of 1º cells
3. incorrect orientation of ommatidial core (likely due to earlier mis-rotation)
4. errors in bristle placement and number
5. incorrect specification of 3º cells
6. additional or missing lattice cells (2ºs + 3ºs)

Table S2. Expected product size for primers used to verify the cnoΔDIL mutant

		Expected product size	
Primer pair		Edited locus	Wild-type locus
1	F. 1 (Forward. 1)	1346 bp	1680 bp
	R. 1 (Reverse. 1)		
2	F. 2 (Forward. 2)	588 bp	4238 bp
	R. 2 (Reverse. 2)		
3	F. 3 (Forward. 3)	No product	1769 bp
	R. 3 (Reverse. 3)		

Table S3. Antibodies used in this study

**Primaries**	**Species**	**Dilution**	**Source**
Anti-Canoe	Rabbit IgG	1:1,000 (IF, WB)	Sawyer et al., 2009
Anti-Bazooka	Rabbit IgG	1:2,000 (IF)	Choi et al., 2013
Anti-Armadillo (N27A1)	Mouse IgG_{2a}	1:100 (IF)	Developmental Studies Hybridoma Bank
Anti-GFP (JL-8)	Mouse IgG_{2a}	1:1,000 (IF, WB)	Clontech Laboratories (632381)
Anti-α-tubulin	Mouse IgG_1	1:5,000 (WB)	Sigma-Aldrich (T6199)
**Secondary antibodies**		**Dilution**	**Source**
Alexa Fluor 488, 568, 647		1:1,000 (IF)	Life Technologies
Anti-Rabbit IRDye 680RD		1:10,000 (WB)	LI-COR Biosciences
Anti-Mouse IRDye 800CW		1:10,000 (WB)	LI-COR Biosciences

IF, immunofluorescence; WB, Western blot.
