# Supplementary material for: The Dilute domain in Canoe is not essential for linking cell junctions to the cytoskeleton but supports morphogenesis robustness
Source: J Cell Sci. 2024 Mar 21;137(6):jcs261734. doi: 10.1242/jcs.261734 (PMC11006394; doi:10.1242/jcs.261734)
Supplement: Supplementary file 1 [file joces-137-261734_review_history.pdf]

The Dilute domain in Canoe is not essential for linking cell junctions to the cytoskeleton but supports morphogenesis robustness

Emily D. McParland, T. Amber Butcher, Noah J. Gurley, Ruth I. Johnson, Kevin C. Slep and Mark Peifer

DOI: 10.1242/jcs.261734

Editor: Kathleen Green

Review timeline

Original submission: 18 October 2023
Editorial decision: 21 November 2023
First revision received: 24 January 2024
Accepted: 29 January 2024

Original submission

<u>First decision letter</u>

MS ID#: JOCES/2023/261734

MS TITLE: The Dilute domain of Canoe is not essential for Canoe's role in linking adherens junctions to the cytoskeleton but contributes to robustness of morphogenesis

AUTHORS: Emily D McParland, T. Amber Butcher, Noah J. Gurley, Ruth I Johnson, Kevin C Slep, and Mark Peifer

ARTICLE TYPE: Research Article

We have now reached a decision on the above manuscript.

To see the reviewers' reports and a copy of this decision letter, please go to: https://submit-jcs.biologists.org and click on the 'Manuscripts with Decisions' queue in the Author Area. (Corresponding author only has access to reviews.)

As you will see, the reviewers are uniformly positive about the work, and two of the three suggest minor revisions only. The third suggested one or two experiments that would strengthen the paper that I would like you to consider.

I would be pleased to see a revised manuscript that addresses the minor and experimental issues raised. I may choose to send the paper back to one of the referees but am open to making the decision myself if you clearly outline revisions, including any experimental additions.

Please ensure that you clearly highlight all changes made in the revised manuscript. Please avoid using 'Tracked changes' in Word files as these are lost in PDF conversion.

I should be grateful if you would also provide a point-by-point response detailing how you have dealt with the points raised by the reviewers in the 'Response to Reviewers' box. Please attend to all of the reviewers' comments. If you do not agree with any of their criticisms or suggestions please explain clearly why this is so.

© 2024. Published by The Company of Biologists under the terms of the Creative Commons Attribution License (https://creativecommons.org/licenses/by/4.0/).

Reviewer 1

Advance summary and potential significance to field

The manuscript by McParland et al. examines the function of the Dilute (DIL) domain of Canoe/Afadin. The structural and functional dissection of adhesion proteins has been an ongoing aim of the adhesion field and the Peifer lab has identified essential and dispensable domains in Canoe/Afadin (Perez-Vale et al 2021), bettering our understanding of the network of protein connections at the adherens junctions and their function. In this study, the authors first use Alpha Fold to predict the structure of the DIL domain and protein interaction partners with this domain. The authors then show that this domain can dimerize and make a deletion mutant to test the function of this domain in Drosophila. They find that the DIL domain is dispensable for viability, supports normal embryonic morphogenesis and localizes normally. Deletion of the DIL domain does have a synergistic effect with reducing Cno levels and the authors show a phenotype for the DIL domain mutant itself in the pupal eye. I found the characterization of the in vivo DIL mutant to be 1) new, 2) straightforward in logic, 3) well quantified and 4) convincing. Overall, this manuscript further illustrates the complexity and multidomain functionality of Canoe, connecting it nicely with past mechanistic work on the function of Canoe in embryogenesis (Sawyer et al. 2009; Sawyer et al. 2011; Bonello et al. 2018; Yu and Zallen 2020) and more generally with the organization of adhesion complexes.

Comments for the author

Specific comments:
1. Fig. 1-3: I realize that Alpha Fold is a relatively new tool and that how it is used in publications is evolving, but I want to bring up my reaction to having three main Results figures showing structures that are predictions. As an experimental cell biologist, my concern is that readers will look at these figures as a results rather than the predictions that they are. One idea is that these figures might be included as supplemental figures (as in Perez-Vale et al. 2021) and that it is explicitly stated that 'interactions' are predicted interactions.
The authors could also report the IDDT score for predictions. I conferred with a structural biologist informally about this and they had a similar reaction to me so I bring it up as part of the discussion.
2. Fig. 9B: Is the difference statistically significant? And error bars should be defined.
3. A point of stylistic preference: the figures are arranged in a haphazard way where panels are different sizes and inconsistent. It would be easier to follow with more streamlined figure style.

Reviewer 2

Advance summary and potential significance to field

A large protein network is emerging in the regulation of the connection between the cytoskeleton and cell-cell junctions. The functional complexity of the network results not only from the number of proteins that can mediate this linkage, but also from the fact that most of these proteins are large multidomain proteins. One such example is Canoe, the fly orthologue of Afadin. Here, McParland et al show a careful and detailed investigation of the function of the Dilute domain of Canoe, through the combination of biochemical studies imaging and Drosophila genetics. This domain is only shared with members with the Myosin V family, but whereas the DIL domain is well characterized for this family, its significance in Canoe/Afadin function is unknown.

Sequence conservation and structural modelling with AlphaFold suggests that the regions that provide many interactions of MyoV are not conserved with the DIL domain of Canoe, whereas biochemical data shows that similar to its function in Myosin V, the Dilute domain of Canoe also promotes its ability to dimerize. This may help explaining the ability of Canoe/Afadin to oligomerize. However, deletion of the DIL domain of Canoe does not affect its localization and function at AJs during embryonic development. In fact, flies lacking this domain are viable of fertile. This likely highlights the robustness of a network created by multidomain proteins and so the authors addressed the importance of this domain in challenging conditions. The authors indeed uncover that this domain could be particularly important if cells are sensitized by a reduction of Canoe protein, and also in challenging morphogenetic events, such as pupal eye development.

© 2024. Published by The Company of Biologists under the terms of the Creative Commons Attribution License (https://creativecommons.org/licenses/by/4.0/).

The work is clearly presented (I particularly commend the authors for an excellent illustration, labelling and description of the structural data, which makes it easier to read for cell biologists) and the conclusions are in line with the evidence provided. The experiments are carefully designed and well quantified (statistical data and methodology is also well described). Overall, the study combines advanced fly genetics with biochemical data and recent access to more precise structural predictions to characterize a protein domain whose importance has been mysterious. Even if it could be surprising that this domain is dispensable for fly viability and fertility, the authors did a great job at providing data showing that ensures the robustness of the Adherens junction network. This manuscript is of interest to a broad community of cell biologists that study the underlaying mechanisms of cell junction organization and tissue architecture. This is a good example of a study characterizing the significance of a specific protein domain in a conserved adherens junction regulator.
Understanding the significance of these domains in multi domain proteins is critical to comprehend the mechanisms that confer robustness to AJ-cytoskeleton linkage, even if many of those may seem redundant in optimal conditions. I recommend publication of the current version of the manuscript almost as it is.

Comments for the author

Minor comment:

Fig. 6; 8K, 8L,10 Q – the error shown in the graph and the statistical test that was used to evaluate significance is well described in the methods, but it would be worth to refer shortly to it in the figure legend.

<u>Reviewer 3</u>

Advance summary and potential significance to field

Overall a nice study that in many ways is broader than a single domain structure/function study of Canoe/Afadin. Indeed, it provides a Â'road-mapÂ' for interrogating the structure and function of multi-domain scaffold proteins with multiple (compensatory) interactions with AJ components to glean fundamental insights, using fly genetics and predictive power of AlphaFold as tools. Authors show that Dilute domain of Canoe contributes to tissue morphogenesis, possibly through favoring Canoe homodimerization.

Comments for the author

SUMMARY: This study seeks to understanding how adherens junctions (AJs) are organize and regulated during embryonic development of flies, focusing on for roles of the large, multi-domain scaffold protein, Canoe/Afadin. Team Peifer has previously shown that Canoe is important for precise coordination of epithelial cell shapes/behaviors required for tissue morphogenesis. Systematic deletion analyses, where Canoe domains (Rap1-GTPase, PDZ, F-actin-binding) are independently removed and mutant variants are restored in flies lacking wild-type Canoe, reveals that two N-terminal Rap1-GTPase domains are most critical to Canoe function, whereas PDZ and F-actin binding domains play less critical roles. The current study provides much needed structural insight and contributing function of a previously poorly characterized region of Canoe/Afadin known as the Dilute (DIL) domain, first characterized in the context of the unconventional myosin, MyoV. The authors leverage AlphaFold to examine predicted structure of the DIL domain for both Canoe and its vertebrate ortholog, Afadin, revealing a 15-a helical structure with potential to engage established, MyoV interactors (e.g., Rab11, MICAL RILPL2). Most intriguingly, the authors identify a large groove with potential to engage Canoe/Afadin-specific ligands, but the identity of such ligands remain unknown. Purification of the Canoe DIL domain reveals its capacity to homodimerize in vitro (SEC-MALS), suggesting a possible role for this domain in context of the full length Canoe. While a CanoeΔDIL mutant does not obviously interfere with basica AJ formation in flies, cell-cell coordination is perturbed in morphogenetically active tissues (e.g., during dorsal closure), demonstrating a clear contributing role. These and other publications from this team suggest that

© 2024. Published by The Company of Biologists under the terms of the Creative Commons Attribution License (https://creativecommons.org/licenses/by/4.0/).

Canoe/Afadin uses multiple structural domains to engage AJ components, where loss of any one component is not critical, likely due to compensatory/redundant localization information. Such interactions are nonetheless important to phenotypic/organismal robustness—perhaps rationalizing conservation of these “non-essential” domains to Canoe/Afadin function across evolution.

ASSESSMENT: Overall a nice study that in many ways is broader than a single domain, structure/function study of Canoe/Afadin. Indeed, it provides a ‘road-map’ for interrogating the structure and function of multi-domain scaffold proteins with multiple (compensatory) interactions with AJ components to glean fundamental insights, using fly genetics and predictive power of AlphaFold as tools. There are, however, a lot of structural possibilities put forward in Fig.3, but the DIL homodimerization model is limited to a validation in a small fragment (Fig. 4).
Can the authors show evidence of Canoe/Afadin homodimerization in context of the full length (or larger fragment) protein? I also have specific questions, which would enhance clarity of the model, and future directions for the field.

Specific Questions:

1. Can the authors test/validate whether the Canoe DIL region mediates homodimerization in the context of full-length Canoe? Can you distinguish WT (untagged Canoe) from CanoeΔDIL if gels are run longer/different % SDS-PAGE to test by immunoprecipitating for GFP-Canoe? If challenging in flies, can the authors use their MDCK system shortened fragments (e.g., Afadin ΔC1 mutant in Fig. 10 of Choi et al., 2016)? It would be nice to have a bit more evidence for the homodimerization model in context of the full length Canoe (or larger fragment)—since the Alpha fold data raise the possibility of other models (heterodimerization between Canoe DIL domain and other proteins that recognize the “groove”).
2. Can the authors show a predicted model for the Canoe DIL homodimer in Alphafold? Or using what is known about the exisiting MyoVb structure as a guide?
3. Is MyoV in the Canoe proteomic space, and might Canoe interact with MyoV via the DIL domain? Evidence the Canoe DIL manifests a major groove not found in MyoV perhaps suggest otherwise, but would be helpful to know how we should think about these potential interactions.
4. Evidence in Fig.3C showing that the MyoV DIL domain can bind a coiled-coil region in another region of MyoV, begs the question of whether the Canoe DIL binds the coiled-coil region of Canoe/Afadin itself? I ask because the loss of DIL appears to enhance the enrichment of Canoe to aligned AP-borders (Fig. 8L). While I recognize such altered localization during convergence-extension can be due to many models (Canoe partner switching due to loss of a DIL-dependent retention mechanism)- it would be helpful to hear the authors thoughts on this.
5. The Canoe PDZ is said to “bind E-cadherin”, but is their evidence for direct binding to the extreme C-terminus of E-cadherin? Or is the direct localization not mapped, and likely due to PDZ-interaction motifs at the C-termini of b-catenin, p120ctn, a-catenin or other interactors such as Bazooka)? Please clarify, as it was not clear from the citations provided (I could not find direct evidence in cited papers).

Minor

6. Authors refer to MyoVb and Myo5b in some places. If indeed referring to the same protein, please unify nomenclature.
7. P.12 sub-title “groove” not grove.

First revision

<u>Author response to reviewers' comments</u>

We are grateful to the reviewers for their generally positive response and thoughtful suggestions regarding our manuscript. We have followed their suggestions, addressing all of the issues by adding new data and making suggested changes in the text and Figures—these are laid out below with our responses in italics. We added new data to both Revised Figure 3 (originally Fig. 4) and Figure S1 (former Figure 2) and clarified revised Figures 5, 7.8. and 9 according to Reviewer suggestions. The text has been revised in multiple places, as laid out in detail below, and the

© 2024. Published by The Company of Biologists under the terms of the Creative Commons Attribution License (https://creativecommons.org/licenses/by/4.0/).

layout of Figures 7,8, and 9 has been refined following a Reviewer suggestion. We also carried out two sets of AlphaFold runs to test suggestions raised by a Reviewer. We also attached a marked-up version of the text with the changes highlighted in red. These revisions have strengthened and extended our work and we are grateful for their suggestions.

Reviewer 1 Advance Summary and Potential Significance to Field:

The manuscript by McParland et al. examines the function of the Dilute (DIL) domain of Canoe/Afadin. The structural and functional dissection of adhesion proteins has been an ongoing aim of the adhesion field and the Peifer lab has identified essential and dispensable domains in Canoe/Afadin (Perez-Vale et al 2021), bettering our understanding of the network of protein connections at the adherens junctions and their function. In this study, the authors first use Alpha Fold to predict the structure of the DIL domain and protein interaction partners with this domain. The authors then show that this domain can dimerize and make a deletion mutant to test the function of this domain in Drosophila. They find that the DIL domain is dispensable for viability, supports normal embryonic morphogenesis and localizes normally. Deletion of the DIL domain does have a synergistic effect with reducing Cno levels and the authors show a phenotype for the DIL domain mutant itself in the pupal eye. I found the characterization of the in vivo DIL mutant to be 1) new, 2) straightforward in logic, 3) well quantified, and 4) convincing. Overall, this manuscript further illustrates the complexity and multidomain functionality of Canoe, connecting it nicely with past mechanistic work on the function of Canoe in embryogenesis (Sawyer et al. 2009; Sawyer et al. 2011; Bonello et al. 2018; Yu and Zallen 2020) and more generally with the organization of adhesion complexes.

Reviewer 1 Comments for the Author:
Specific comments:
1. Fig. 1-3: I realize that Alpha Fold is a relatively new tool and that how it is used in publications is evolving, but I want to bring up my reaction to having three main Results figures showing structures that are predictions. As an experimental cell biologist, my concern is that readers will look at these figures as a results rather than the predictions that they are. One idea is that these figures might be included as supplemental figures (as in Perez-Vale et al. 2021) and that it is explicitly stated that 'interactions' are predicted interactions.
The authors could also report the IDDT score for predictions. I conferred with a structural biologist informally about this and they had a similar reaction to me, so I bring it up as part of the discussion.
We agree with the reviewer and have moved the original Fig. 2 to the Supplemental Material (now Fig. S1). We have renumbered figures accordingly. We also now report the confidence of the AlphaFold Cno and Afadin DIL structural models using pLDDT values graphically in Fig. S1A-B, and compare this to the experimentally determined B-factors from the reported MyoVb DIL crystal structure (Fig. S1C) (Nascimento et al., 2013). We also report the overall pLDDT value range and Ca atom average for both AlphaFold DIL structural models in the text as follows: "(Cno DIL pLDDT range: 99-62, Ca average: 93; Afadin DIL pLDDT range: 97-49, Ca average: 90)." When describing the structure and its intramolecular interactions, we now specify that these are predicted interactions.

2. Fig. 9B: Is the difference statistically significant? And error bars should be defined.
Sorry for the lack of clarity here. To be conservative, rather than calculating statistical tests using the number of embryos analyzed (n>1250), we chose to calculate it based on the average lethality in each experiment (n=8). We have revised the Figure to replace that panel, including dots illustrating the lethality observed in each experiment. The mean and standard deviation are indicated. When analyzed using Welch's t test using n=8 suggested the difference in lethality was not significant (p=0.15). This is now explained more clearly in both the text and the Figure lend.

3. A point of stylistic preference: the figures are arranged in a haphazard way where panels are different sizes and inconsistent. It would be easier to follow with more streamlined figure style.

We have edited Figures 7, 8, and 9 to reduce this issue.

© 2024. Published by The Company of Biologists under the terms of the Creative Commons Attribution License (https://creativecommons.org/licenses/by/4.0/).

*****

Reviewer 2 Advance Summary and Potential Significance to Field:

A large protein network is emerging in the regulation of the connection between the cytoskeleton and cell-cell junctions. The functional complexity of the network results not only from the number of proteins that can mediate this linkage, but also from the fact that most of these proteins are large multidomain proteins. One such example is Canoe, the fly orthologue of Afadin. Here, McParland et al show a careful and detailed investigation of the function of the Dilute domain of Canoe, through the combination of biochemical studies, imaging and Drosophila genetics. This domain is only shared with members with the Myosin V family, but whereas the DIL domain is well characterized for this family, its significance in Canoe/Afadin function is unknown.

Sequence conservation and structural modelling with AlphaFold suggests that the regions that provide many interactions of MyoV are not conserved with the DIL domain of Canoe, whereas biochemical data shows that similar to its function in Myosin V, the Dilute domain of Canoe also promotes its ability to dimerize. This may help explaining the ability of Canoe/Afadin to oligomerize. However, deletion of the DIL domain of Canoe does not affect its localization and function at AJs during embryonic development. In fact, flies lacking this domain are viable of fertile. This likely highlights the robustness of a network created by multidomain proteins and so the authors addressed the importance of this domain in challenging conditions. The authors indeed uncover that this domain could be particularly important if cells are sensitized by a reduction of Canoe protein, and also in challenging morphogenetic events, such as pupal eye development.

The work is clearly presented (I particularly commend the authors for an excellent illustration, labelling and description of the structural data, which makes it easier to read for cell biologists) and the conclusions are in line with the evidence provided. The experiments are carefully designed and well quantified (statistical data and methodology is also well described). Overall, the study combines advanced fly genetics with biochemical data and recent access to more precise structural predictions to characterize a protein domain whose importance has been mysterious. Even if it could be surprising that this domain is dispensable for fly viability and fertility, the authors did a great job at providing data showing that ensures the robustness of the Adherens junction network. This manuscript is of interest to a broad community of cell biologists that study the underlaying mechanisms of cell junction organization and tissue architecture. This is a good example of a study characterizing the significance of a specific protein domain in a conserved adherens junction regulator. Understanding the significance of these domains in multi domain proteins is critical to comprehend the mechanisms that confer robustness to AJ-cytoskeleton linkage, even if many of those may seem redundant in optimal conditions. I recommend publication of the current version of the manuscript almost as it is.

We are grateful to you for your favorable comments.

Reviewer 2 Comments for the Author:
Minor comment:

Fig. 6; 8K, 8L,10 Q – the error shown in the graph and the statistical test that was used to evaluate significance is well described in the methods, but it would be worth to refer shortly to it in the figure legend.

Added—thanks for the pointer.

*****

Reviewer 3 Advance summary and potential significance to field

Overall a nice study that in many ways is broader than a single domain, structure/function study of Canoe/Afadin. Indeed, it provides a ‘road-map’ for interrogating the structure and function of multi-domain scaffold proteins with multiple (compensatory) interactions with AJ components to glean fundamental insights, using fly genetics and predictive power of AlphaFold as tools. Authors show that Dilute domain of Canoe contributes to tissue morphogenesis, possibly through favoring Canoe homodimerization.

© 2024. Published by The Company of Biologists under the terms of the Creative Commons Attribution License (https://creativecommons.org/licenses/by/4.0/).

Reviewer 3 Comments for the author

SUMMARY: This study seeks to understanding how adherens junctions (AJs) are organize and regulated during embryonic development of flies, focusing on for roles of the large, multi-domain scaffold protein, Canoe/Afadin. Team Peifer has previously shown that Canoe is important for precise coordination of epithelial cell shapes/behaviors required for tissue morphogenesis. Systematic deletion analyses, where Canoe domains (Rap1-GTPase, PDZ, F-actin-binding) are independently removed and mutant variants are restored in flies lacking wild-type Canoe, reveals that two N-terminal Rap1-GTPase domains are most critical to Canoe function, whereas PDZ and F-actin binding domains play less critical roles. The current study provides much needed structural insight and contributing function of a previously poorly characterized region of Canoe/Afadin known as the Dilute (DIL) domain, first characterized in the context of the unconventional myosin, MyoV. The authors leverage AlphaFold to examine predicted structure of the DIL domain for both Canoe and its vertebrate ortholog, Afadin, revealing a 15-a helical structure with potential to engage established, MyoV interactors (e.g., Rab11, MICAL, RILPL2). Most intriguingly, the authors identify a large groove with potential to engage Canoe/Afadin-specific ligands, but the identity of such ligands remain unknown. Purification of the Canoe DIL domain reveals its capacity to homodimerize in vitro (SEC-MALS), suggesting a possible role for this domain in context of the full length Canoe. While a CanoeΔDIL mutant does not obviously interfere with basic AJ formation in flies, cell-cell coordination is perturbed in morphogenetically active tissues (e.g., during dorsal closure), demonstrating a clear contributing role. These and other publications from this team suggest that Canoe/Afadin uses multiple structural domains to engage AJ components, where loss of any one component is not critical, likely due to compensatory/redundant localization information. Such interactions are nonetheless important to phenotypic/organismal robustness—perhaps rationalizing conservation of these “non- essential” domains to Canoe/Afadin function across evolution.

ASSESSMENT: Overall a nice study that in many ways is broader than a single domain, structure/function study of Canoe/Afadin. Indeed, it provides a ‘road-map’ for interrogating the structure and function of multi-domain scaffold proteins with multiple (compensatory) interactions with AJ components to glean fundamental insights, using fly genetics and predictive power of AlphaFold as tools. There are, however, a lot of structural possibilities put forward in Fig.3, but the DIL homodimerization model is limited to a validation in a small fragment (Fig. 4). Can the authors show evidence of Canoe/Afadin homodimerization in context of the full length (or larger fragment) protein? I also have specific questions, which would enhance clarity of the model, and future directions for the field.

Specific Questions:

1. the authors test/validate whether the Canoe DIL region mediates homodimerization in the context of full-length Canoe? Can you distinguish WT (untagged Canoe) from CanoeΔDIL if gels are run longer/different % SDS-PAGE to test by immunoprecipitating for GFP-Canoe? If challenging in flies, can the authors use their MDCK system shortened fragments (e.g., Afadin ΔC1 mutant in Fig. 10 of Choi et al., 2016)? It would be nice to have a bit more evidence for the homodimerization model in context of the full length Canoe (or larger fragment)— since the Alpha fold data raise the possibility of other models (heterodimerization between Canoe DIL domain and other proteins that recognize the “groove”).

We agree this is an important question. Unfortunately the addition of the GFP-tag and the deletion of the Dilute domain essentially cancel on another out, and both proteins are recognized by our anti-Canoe Antibody, so the experiment in flies is not possible. Instead, we have asked whether a longer protein encompassing the FHA, Dilute and PDZ domains also dimerized. It did so, albeit at a lower frequency. These data are included in a revised Figure 3. We also have added caveats to that section of the results and to the relevant section of the Discussion as follows: “To determine if dimerization was retained in constructs carrying additional folded domains, we cloned a construct extending from the beginning of the Cno FHA domain to the end of the PDZ domain (FHA-DIL-PDZ), expressed this in E. coli and purified it (Fig. 3C). This construct also migrated as both a monomer and a dimer (Fig. 3D), though the dimer fraction was lower. It will be important in the future to explore potential roles for the DIL domain in the oligomerization we and others have observed in vivo.” AND “It will be important in the future to explore potential roles for the DIL domain in the oligomerization we and others have observed in vivo.”

© 2024. Published by The Company of Biologists under the terms of the Creative Commons Attribution License (https://creativecommons.org/licenses/by/4.0/).

2. Can the authors show a predicted model for the Canoe DIL homodimer in Alphafold? Or using what is known about the existing MyoVb structure as a guide?

This is an interesting idea. Prompted by this suggestion, we performed a number of AlphaFold runs using two Cno DIL domains as input, but the top output models all varied in the way that the domains were positioned relative to one another. When we examined each model, the number of potential interactions at the assorted dimer interfaces was low, and these potential interactions were often not complementary. Putting this together, we do not think that AlphaFold was able to predict the homodimeric state that we detected in vitro. Our long-term goal is to explore Canoe structure using cryo-EM, which will help address this question, especially if there are different structural states of Canoe (e.g. monomer and homodimer).

Is MyoV in the Canoe proteomic space, and might Canoe interact with MyoV via the DIL domain? Evidence the Canoe DIL manifests a major groove not found in MyoV perhaps suggest otherwise, but would be helpful to know how we should think about these potential interactions.

This is an interesting idea. MyoV is NOT on the lists included in two previously published proximity labeling experiments using BioID fused to mammalian Afadin as bait [https://pubmed.ncbi.nlm.nih.gov/35931706/ and https://pubmed.ncbi.nlm.nih.gov/34493720/]. We have unpublished BioID data from our lab and this includes only Myo 1, not MyoV. Given the negative result with its caveats, we don't think adding this information would be very informative.

4. Evidence in Fig.3C showing that the MyoV DIL domain can bind a coiled-coil region in another region of MyoV, begs the question of whether the Canoe DIL binds the coiled-coil region of Canoe/Afadin itself? I ask because the loss of DIL appears to enhance the enrichment of Canoe to aligned AP-borders (Fig. 8L). While I recognize such altered localization during convergence-extension can be due to many models (Canoe partner switching due to loss of a DIL-dependent retention mechanism)- it would be helpful to hear the authors thoughts on this.

This is another interesting question, and prompted by it we tried multiple AlphaFold runs to see if the program could afford any predictive insight into potential Dilute domain/IDR interactions.. When we ran AlphaFold using the DIL domain and the IDR (which contains predicted helical segments in its middle, and at its C-terminal region) in trans, no specific, consistent interaction was detected. This negative modeling result aligns with AlphaFold's predicted structure of full-length Canoe, for which no definitive interaction between the DIL domain and the IDR was predicted. We next inquired if any of the IDR helical regions, if input as two copies, could themselves homodimerize, and in a homodimeric state bind to the DIL domain. AlphaFold model predictions did not have any of the IDR helical regions homodimerizing – they were all monomeric, and in their monomeric states, they did not interact with the DIL domain. As noted above, there are extensive lists of proteins identified as being proximal in vivo to Canoe/afadin. One of our goals moving forward is to use AlphaFold to explore these candidates for predicted interactions with Canoe's DIL domain, and then to test these interactions using biochemical and genetic approaches. We have now noted this in the Discussion.

5. The Canoe PDZ is said to "bind E-cadherin", but is their evidence for direct binding to the extreme C-terminus of E-cadherin? Or is the direct localization not mapped, and likely due to PDZ-interaction motifs at the C-termini of b-catenin, p120ctn, a-catenin or other interactors such as Bazooka)? Please clarify, as it was not clear from the citations provided (I could not find direct evidence in cited papers).

Our apologies for lack of clarity—we had lumped references supporting interactions with E-cadherin and nectins after mentioning the nectin interaction. We provided evidence that the C-terminal peptide from E-cadherin can bind the Canoe PDZ domain using purified proteins (Sawyer et al. 2009). We now have rearranged the sentence to make this clearer "The central PDZ domain binds several partners, including the transmembrane junctional proteins E-cadherin (Ecad; (Sawyer et al., 2009)) and Nectins/Echinoid (Takahashi et al., 1999; Wei et al., 2005)."

Minor

© 2024. Published by The Company of Biologists under the terms of the Creative Commons Attribution License (https://creativecommons.org/licenses/by/4.0/).

6. Authors refer to MyoVb and Myo5b in some places. If indeed referring to the same protein, please unify nomenclature.

Fixed—thank you!

7.P.12 sub-title “groove” not grove.

Big oops-- Fixed—thank you!

Second decision letter

MS ID#: JOCES/2023/261734

MS TITLE: The Dilute domain of Canoe is not essential for Canoe's role in linking adherens junctions to the cytoskeleton but contributes to robustness of morphogenesis

AUTHORS: Emily D McParland, T. Amber Butcher, Noah J. Gurley, Ruth I Johnson, Kevin C Slep, and Mark Peifer

ARTICLE TYPE: Research Article

I am happy to tell you that your manuscript has been accepted for publication in Journal of Cell Science, pending standard ethics checks.

© 2024. Published by The Company of Biologists under the terms of the Creative Commons Attribution License (https://creativecommons.org/licenses/by/4.0/).
